# Fast functional mapping of ligand-gated ion channels

**DOI:** 10.1038/s42003-023-05340-w

**Published:** 2023-10-02

**Authors:** Ralf Schmauder, Thomas Eick, Eckhard Schulz, Günther Sammler, Elmar Voigt, Günter Mayer, Holger Ginter, Günter Ditze, Klaus Benndorf

**Affiliations:** 1 Institut für Physiologie II, Universitätsklinikum Jena, Friedrich-Schiller-Universität Jena, 07743 Jena, Germany; 2 Hochschule Schmalkalden, Fakultät Elektrotechnik, Blechhammer, 98574 Schmalkalden, Germany; 3 Zentrale Forschungswerkstätten, Universitätsklinikum Jena, Friedrich-Schiller-Universität Jena, 07743 Jena, Germany; 4 Leibniz Institut für Photonische Technologien e.V., Albert-Einstein-Straße 9, 07745 Jena, Germany

**Keywords:** Patch clamp, Kinetics, Ion transport, Perturbations, Thermodynamics

## Abstract

Ligand-gated ion channels are formed by three to five subunits that control the opening of the pore in a cooperative fashion. We developed a microfluidic chip-based technique for studying ion currents and fluorescence signals in either excised membrane patches or whole cells to measure activation and deactivation kinetics of the channels as well as ligand binding and unbinding when using confocal patch-clamp fluorometry. We show how this approach produces in a few seconds either unidirectional concentration-activation relationships at or near equilibrium and, moreover, respective time courses of activation and deactivation for a large number of freely designed steps of the ligand concentration. The short measuring period strongly minimizes the contribution of disturbing superimposing effects such as run-down phenomena and desensitization effects. To validate gating mechanisms, complex kinetic schemes are quantified without the requirement to have data at equilibrium. The new method has potential for functionally analyzing any ligand-gated ion channel and, beyond, also for other receptors.

## Abstract (web summary)

A multi-channel solution exchange system supplies up to 30 different external solutions to ligand-gated ion channels in a membrane patch in rapid succession, allowing to determine detailed dose-response relationships and dynamics in seconds.

## Introduction

Ligand-gated ion channels are integral membrane proteins with the ability to bind small soluble ligand molecules and to translate this binding signal to a changed ion conductance across the membrane. Structurally, ligand-gated ion channels are oligomers composed of three to five subunits comprising a defined number of ligand binding sites and a conjoint pore to conduct the ions. Such a multimeric architecture of the channels enables cooperative action of their subunits, allowing for finely tuned cellular responses to ligand signals. When ligands bind to these binding sites, the open probability of the pore can be either increased, in the case of agonistic ligands, or decreased, in the case of antagonistic ligands.

The most common approach to understanding the cooperativity of the subunits in a ligand-gated ion channel by an agonist is to determine a so-called concentration–activation (dose–response) relationship at equilibrium. To this end, the amplitude of the ionic current, measured by an appropriate electrophysiological technique, is determined at pre-defined ligand concentrations, normalized with respect to the maximum current, and plotted as a function of the ligand concentration to be fitted by the Hill equation as simple evaluation, providing a concentration of half-maximum activation (EC_50_) and a Hill coefficient (*H*; see textbooks of pharmacology). However, despite the widespread use of this type of analysis, it is severely limited by four reasons: (1) The Hill equation assumes that the ligands bind exactly at the same time, which is physically very unlikely. (2) True equilibria are often not achieved under real experimental conditions with finite duration due to superimposing slow processes, e.g. reversible desensitization or irreversible run down. (3) Any analysis at equilibrium provides per se no information about rates of the underlying processes. (4) The co-existence of positive and negative cooperativity^1,2^ is ignored.

To gain kinetic information about the activation of ligand-gated ion channels, non-equilibrium approaches were performed by combining the patch-clamp technique with the method of concentration jumps, and analyzing the data in terms of Markov models^2–5^. Defined rapid concentration jumps for switching a ligand concentration on and off are typically realized by piezo devices moving a double-barreled glass pipette with two parallel laminar fluid streams^6,7^. Theoretically, for outside-out patches positioned at the very tip of the patch pipette, this approach has a speed limit for the jumps of the ligand concentration at 20 μs^8^, though such short times require critically high flow rates and are practically hardly reached^9^. Typical switching times are in the range of several hundred microseconds^10^. For recording from whole cells, only slower flow rates can be used to keep the cell at the pipette tip functionally intact. In inside-out patches, used to study ion channels gated by intracellular ligands, the speed of the concentration jumps is additionally decreased by ligand diffusion between the pipette tip and the patch within the pipette lumen and, moreover, intracellular materials sticking at the cytosolic face of the patch can further slow diffusion^11^. Consequently, concentration exchanges are not instantaneous steps, but last milliseconds or even longer. As a consequence, any analyses of activation by kinetic schemes are limited by the speed of the concentration jumps.

We present a concentration-mapping method that provides a significant advantage to study ligand-gated ion channels under outside-out, inside-out, and whole-cell patch conditions by applying multiple concentration jumps. The power of the method is demonstrated for the activation of trimeric purinergic P2X2 channels that are activated by extracellular ATP^12–14^ and for tetrameric olfactory CNGA2 channels that are activated by intracellular cyclic nucleotides^15–20^. Moreover, we combine the current recordings with parallel optical recordings of the binding of fluorescent agonists by using confocal patch-clamp fluorometry (cPCF)^1,2^.

Our complex concentration mapping method allows an investigator to derive information about fast and slow gating processes and minimizes run-down or desensitization effects. Moreover, the speed of the actual ligand concentration jumps can be taken into account to improve the validation of kinetic gating schemes. Our method can be used either to build full equilibrium (or quasi-equilibrium) concentration–activation or concentration–binding relationships for up to 25 ligand concentrations or to generate freely designable complex concentration protocols, all in a couple of seconds. For the analysis of the data by kinetic schemes, a true equilibrium is not required.

## Results

### The 30-barrel microfluidic device

**Fig. 1:**
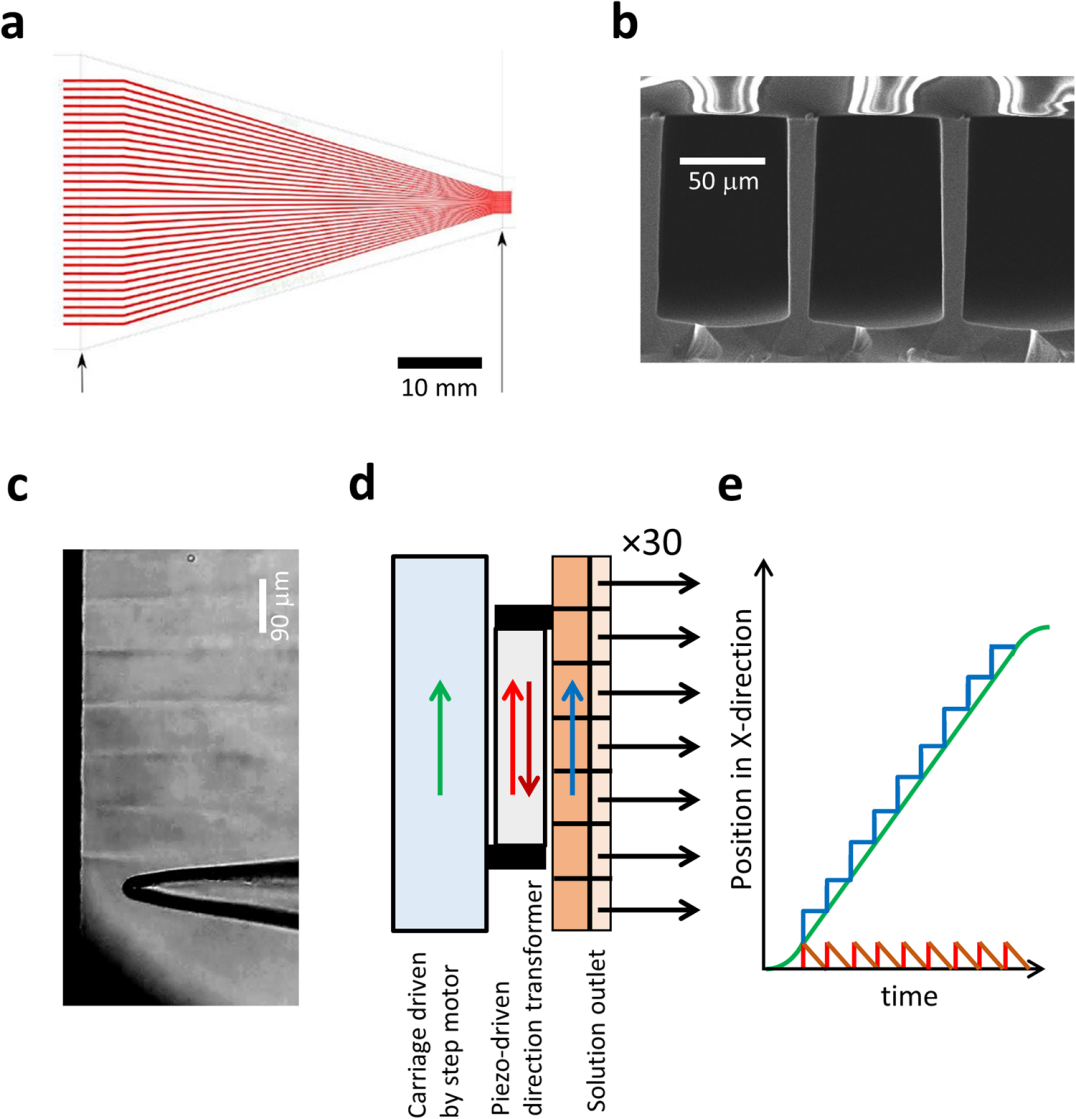
Microfluidic device (MFD) and the multi-channel solution applicator (MCSA). **a** Schematic of the whole MFD showing the 30 barrels in red color arranged in a plane. The MFD was cut and polished at the site indicated by the arrow. **b** Scanning-electron micrograph of the MFD outlet. The barrels had an approximately rectangular cross-section. **c** Laminar flow of solutions into the bath chamber of seven out of 30 barrels. The contrast between the streams is due to refractive index differences between alternating KCl concentrations (120 and 150 mM) in the solutions. **d** Movement of the components of the MFD. An encoded translation stage moved with constant speed in the *X*-direction (green arrow). A piezo and a direction transformer were mounted on the translation stage such that it caused an additional shift in the *X*-direction depending on the controlling voltage (red and brown arrow). The MFD was mounted on the direction transformer in a way that the solution flow out of the barrels (black arrows) was exactly perpendicular to the *X*-direction. **e** Time relationships of the different movements for the example of forward direction according to the arrow colors in (**d**). The coordinated motions of the translation stage and the piezo-controlled direction transformer realized a step-like movement of the outlets such that the patch pipette could be subjected to 26 solution jumps in one run. Each piezo step covered 90 µm, and a total of 3 mm is covered in the *X*-direction. The patch pipette stayed between 100 and 2000 ms in each solution.

The basic idea of our strategy was to extend the idea of a free-standing double-barreled theta glass pipette, providing laminar solution streams at the tip of a patch pipette^6^, to an also free-standing applicator containing a large number of microfluidic channels. A respective microfluidic device (MFD) was manufactured by a silicon-based microstructuring technique in which 30 channels were arranged in a planar manner (Fig. 1a). The outlets had an approximately rectangular shape with a height and width of 120 and 78 μm, respectively (Fig. 1b) and 90 µm periodicity. The MFD was connected to 30 separate solution reservoirs via a parallel silicon connector and thin PTFE tubes with an internal diameter of 300 μm. The solution flow through the microfluidic channels was driven by gravity and set to 27 μl/min, resulting in a speed of 70 mm s^−1^ at the outlets. The flow of the 30 solutions was switched on and off by 30 electronic microvalves. Under these conditions, robust and stable parallel laminar solution streams in the bath solution were reached, extending over several 100 µm away from the outlets (Fig. 1c).

### The operating principle of the multi-channel solution applicator

Analog to rapid solutions switches with theta glass pipettes^6,7^, concentration jumps between adjacent barrels were produced by a piezo actuator (maximum length expansion 120 μm, Physik Instrumente P-212.80). Due to technical constraints, the length expansion of the piezo actuator was transformed from vertical into *x*-direction parallel to the sequence of barrels (Fig. 1d). The MFD was mounted on a lever attached to the direction transformer using leverage of two. The piezo actuator was mounted on a precision linear translation stage driven by a DC motor under encoder-based closed-loop control (Physik Instrumente M-404.1PD). A computer program controlled the piezo actuator, the sliding carriage and the micro-valves. The whole system is termed in the following multi-channel solution applicator (MCSA).

The 30 outlets were used in the following way: Outlet 1, test solution for channel expression; Outlets 2 and 3, initial ligand concentration; Outlets 4–28, test solutions with the desired ligand concentrations; Outlets 29 and 30, initial ligand concentration for reverse run. These pairs of outlets ensured constant conditions for the acceleration/deceleration phases of the DC-stage movements. At the beginning of the experiment, the patch pipette was set to the center of the first measuring position (outlet 4) with a solution containing 0.5–1 µM fluorescein, facilitating positioning of the pipette, particularly in *Z*-dimension, and monitoring a regular flow profile. In *Y*-direction, the direction of the solution flow, we positioned the tip of the patch pipette less than 50 μm away from the outlets. Several seconds before a measurement, the flow of all solutions was started. To achieve constant velocity of the translation stage when starting the measurement at outlet 4, the translation stage was first moved back to the position where the patch opposed outlet 2 and accelerated from there (Fig. 1e). To keep the patch in the middle of this outlet for a desired time interval, the piezo contracts to compensate exactly for the speed of the translation stage. To switch to the next outlet, the piezo skipped forward in the *X*-direction to the next outlet and started contracting again. This coordinated operation of the continuously moving translation stage and the piezo actuator allowed us to skip directly into the middle of all outlets of the MFD in the sequence of their position. Notably, the fast movement of the solution interfaces facilitated by the piezo also improved the precision of the time points of solution exchange in case of slightly varying flow boundaries at the outlets. Vibrational artefacts in the recordings could be avoided by lowering the speed of the piezo and using a time constant for the piezo movement of 3 ms (Supplementary Fig. 1). It should be noted that the actual trajectory through the solution interface is much shorter, typically below 1 ms.

The speed of the translation stage and the compensation movement by the piezo actuator could be coordinated such that the dwell times of the patch pipette in front of an outlet could be varied over a wide range.

### Concentration–activation relationships obtained with the MCSA either at or close to equilibrium

When determining classical concentration–activation (dose–response) relationships either at or close to equilibrium, the individual concentration pulses should be chosen long compared to the slowest activation and deactivation components.

**Fig. 2:**
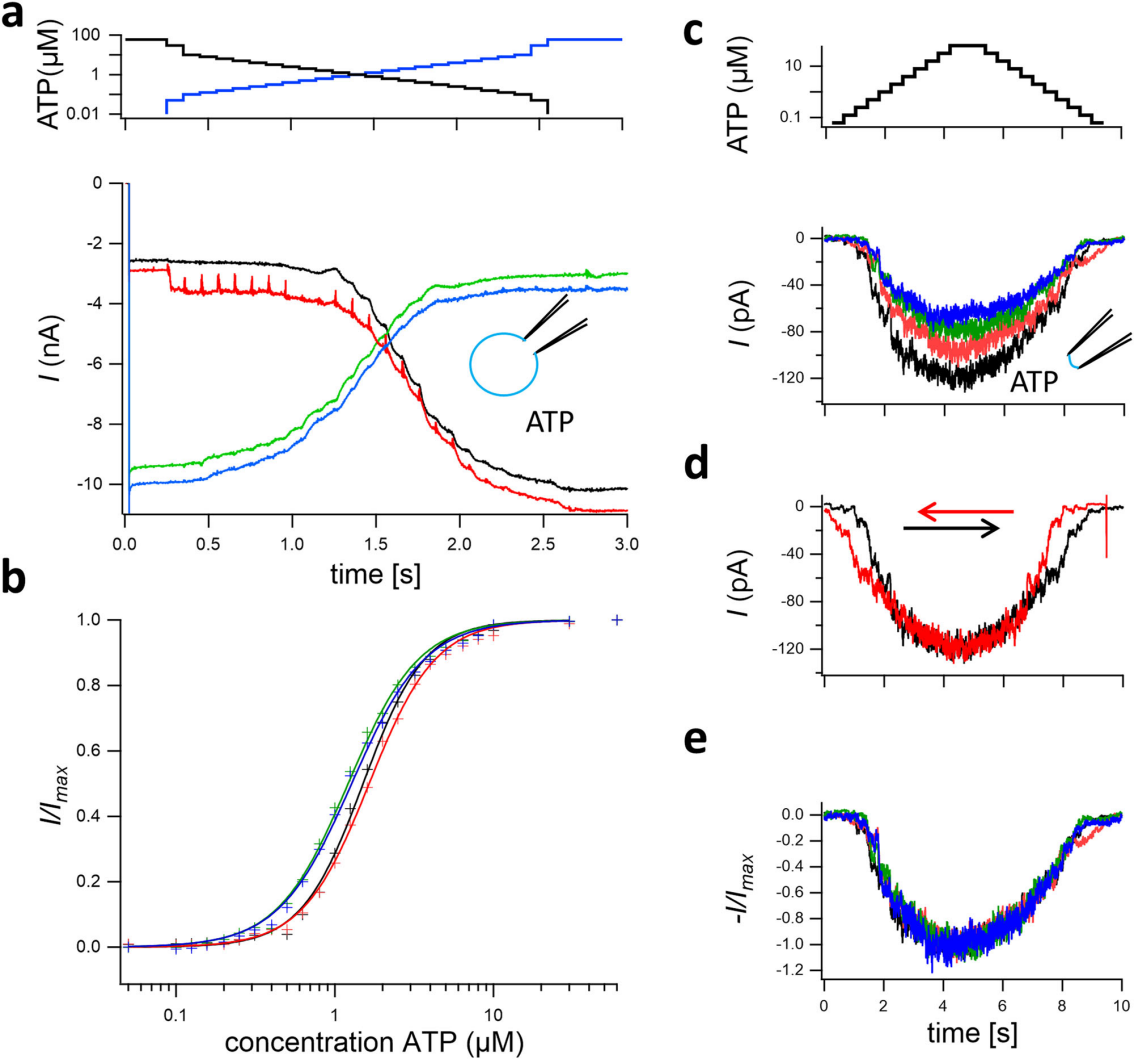
Concentration–activation relationships in P2X2 receptors. **a** P2X2 currents in a whole HEK293 cell at 25 incremental (black) and 25 decremental (green) ATP concentrations (top) with a pulse duration of 100 ms each. Holding potential −50 mV. Time between increasing and decreasing concentration runs 7 s, time between both pulse series 14 s. Red and blue lines are repeated measurements after several minutes and deteriorating seal quality. **b** Concentration–activation relationships of the late currents (average of last 0.6 ms at each pulse) for a first (black, green) and second (red, blue) incremental and decremental pulse sequence, the deteriorating seal did not strongly affect the dynamics. The fit results were EC_50_ = 1.50 ± 0.02 µM, *H* = 2.23 ± 0.07 for increasing and EC_50_ = 1.21 ± 0.02 µM, *H* = 1.95 ± 0.05 for decreasing initial runs and EC_50_ = 1.65 ± 0.02 µM, *H* = 2.08 ± 0.04 for increasing and EC_50_ = 1.28 ± 0.02 µM, *H* = 1.89 ± 0.05 for decreasing runs upon repetition with some sign of patch deterioration. **c** Four recordings of P2X2 currents in an outside-out patch from a HEK293 cell evoked by staircases of 12 incremental and 12 decremental ATP concentrations (top) each. The sequence in the recordings was black, red, green, and blue. Pulse duration 500 ms, holding potential −50 mV, time between pulse series 15 s. Concentration series and time scales are common for panels (**c**–**e**). **d** Normalized current traces from (**c**). **e** Superimposition of the black current trace in **c** with its mirrored trace (red). The asymmetry reveals slow gating which is still incomplete at the end of the individual 500-ms pulses.

We first consider data of whole-cell currents of P2X2 channels expressed in HEK293 cells (Fig. 2a). In the case of P2X2 channels expressed in HEK293 cells, we applied a staircase of 25 ATP steps of 100 ms duration in an equidistant manner on a logarithmic scale, first with incremental and then with decremental concentrations. The resulting whole-cell currents as a function of time reveal a systematic current increase and decrease, respectively. The activation and deactivation kinetics for the individual concentration steps remain detectable. It should be emphasized that recording such full concentration–activation relationships required only the short time interval of 25 × 0.1 = 2.5 s. This minimizes the risk of leakage changes as well as other slow time-dependent changes reported for P2X channels to appear in tens of seconds, including desensitization^21^ and any other run-down phenomena. The corresponding concentration–activation relationships, determined from the amplitude of a 5 ms interval at the end of each pulse, show the characteristic sigmoidal shape (black relationship in Fig. 2b). A second result is that the relationship determined by the decremental concentration sequence (green) is shifted to a small extent to lower concentrations with respect to the incremental concentration staircase. This kind of hysteresis indicates that at the end of the 100 ms concentration pulses, a true equilibrium for the gating was not reached. Repeating the experiment after a couple of seconds showed that the hysteresis is reproducible (red and blue relationships in Fig. 2a and b), even after a noticeable change of the leak current as indicated by the amplitude difference in the current recordings (Fig. 2a).

We next show data from outside-out patches of P2X2 channels obtained from the HEK293 cells to address the phenomenon of run down and to demonstrate how powerful the feature to record concentration–activation relationships in seconds is indeed. We applied four staircases consisting of 12 incrementing and 12 decrementing ATP pulses of 500 ms duration each. These staircases caused a respective increase and decrease of the current amplitude. While the run down (a progressive decrease of current amplitude) within one concentration series was low, repetition of the experiment revealed a rundown on a slower time scale (Fig. 2c). Normalizing these signals revealed that the reduction of current amplitude did not affect the change of the current pattern (Fig. 2e). Thus, the run down due to either silencing of channels or a loss of the patch surface did not alter the channel properties. A further result is that also concentration pulses of 400 ms duration did not suffice to generate a true equilibrium in the gating. This becomes directly evident from the original data by mirroring the current signal of the first ATP sequence with respect to the maximum amplitude (Fig. 2d).

**Fig. 3:**
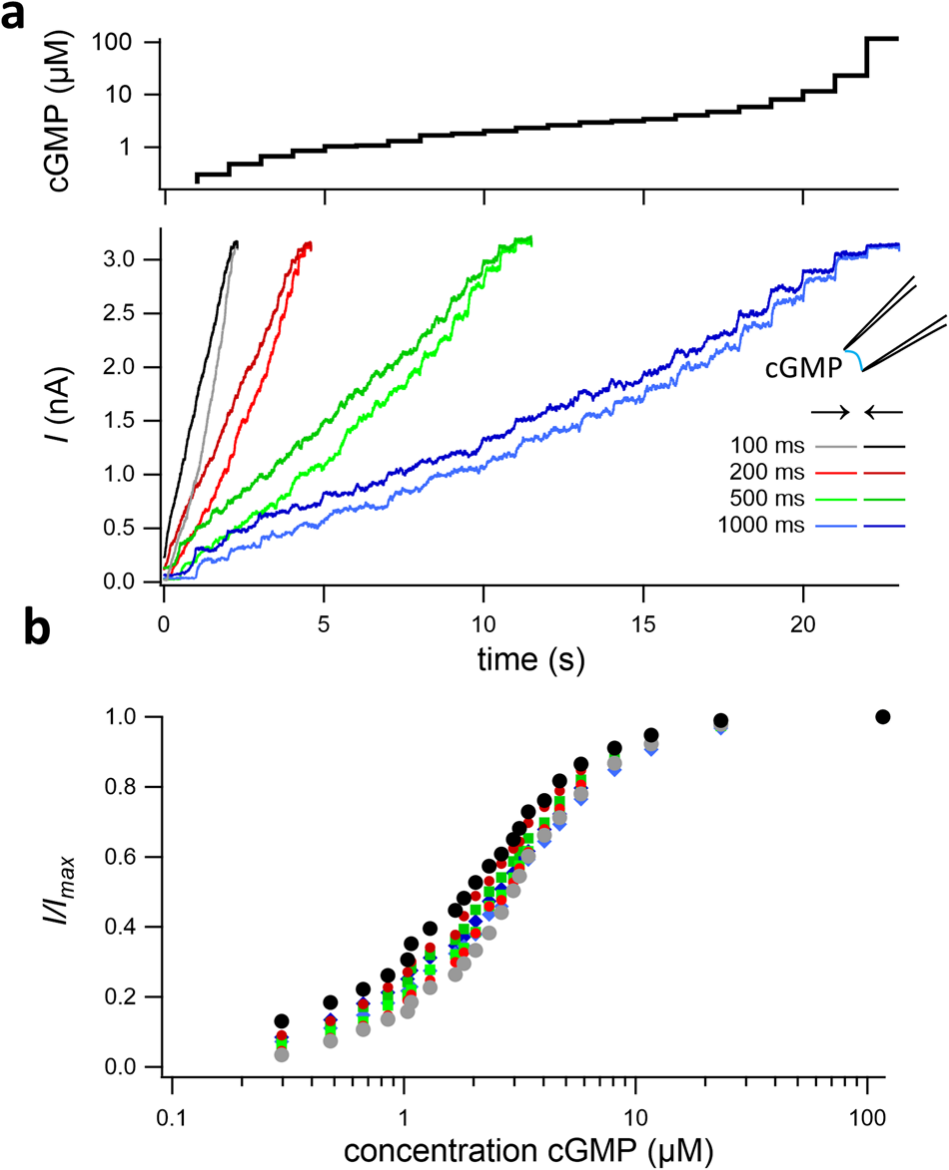
Ligand binding and activation in CNGA2 channels at or close to equilibrium. **a** In inside-out patches of *Xenopus* oocytes containing CNGA2 channels, the channels were activated by cGMP in the bath solution by 23 consecutive steps that were estimated to result in approximately equal response steps (top diagram) according to the known EC_50_ and Hill coefficient^44^. The voltage was +50 mV. Current recordings at the three-step durations 100, 200, 500, and 1000 ms (bottom diagram). The concentration steps were passed in both directions, incremental (light colors) and decremental (dark colors). **b** Concentration–activation relationships for the late current amplitude of the concentration steps in (**a**). Hysteresis was observed for all durations of the concentration steps, indicating that during the recording a true equilibrium was not reached. See Supplementary Fig. 2a–c for fits.

We next demonstrate the application of our method to describe the activation of homotetrameric cyclic nucleotide-gated channels incorporated in inside-out patches. These inside-out patches were obtained from *Xenopus* oocytes expressing these channels. The channels were activated by systematically increasing and decreasing the cGMP concentration, again in an equidistant manner on a logarithmic scale (Fig. 3a). These two staircases were applied with four pulse durations of the individual steps, 100, 200, 500, and 1000 ms. Hysteresis is immediately visible for all step durations and also in the corresponding concentration–activation relationships, determined from the current amplitude of the last 5% of the intervals at the end of each pulse (Fig. 3b). As for P2X2 receptors, this hysteresis indicates that during the recording a true equilibrium was not reached. Note also that the divergence between increasing and decreasing runs reduces with increased pulse length. Corresponding fits with the Hill-equation (Supplementary Fig. 2a–c) and a plot with scaled maximum currents (Supplementary Fig. 2d) exemplifies that channel dynamics and not solution application is the cause of the hysteresis.

Together, these data show that our application system with the developed MFD can easily produce concentration–activation relationships with up to 25 data points within a few seconds. This allows an investigator to minimize the overlay of run-down phenomena or changes in the leakage.

### Relating ligand binding to activation gating with the MCSA

**Fig. 4:**
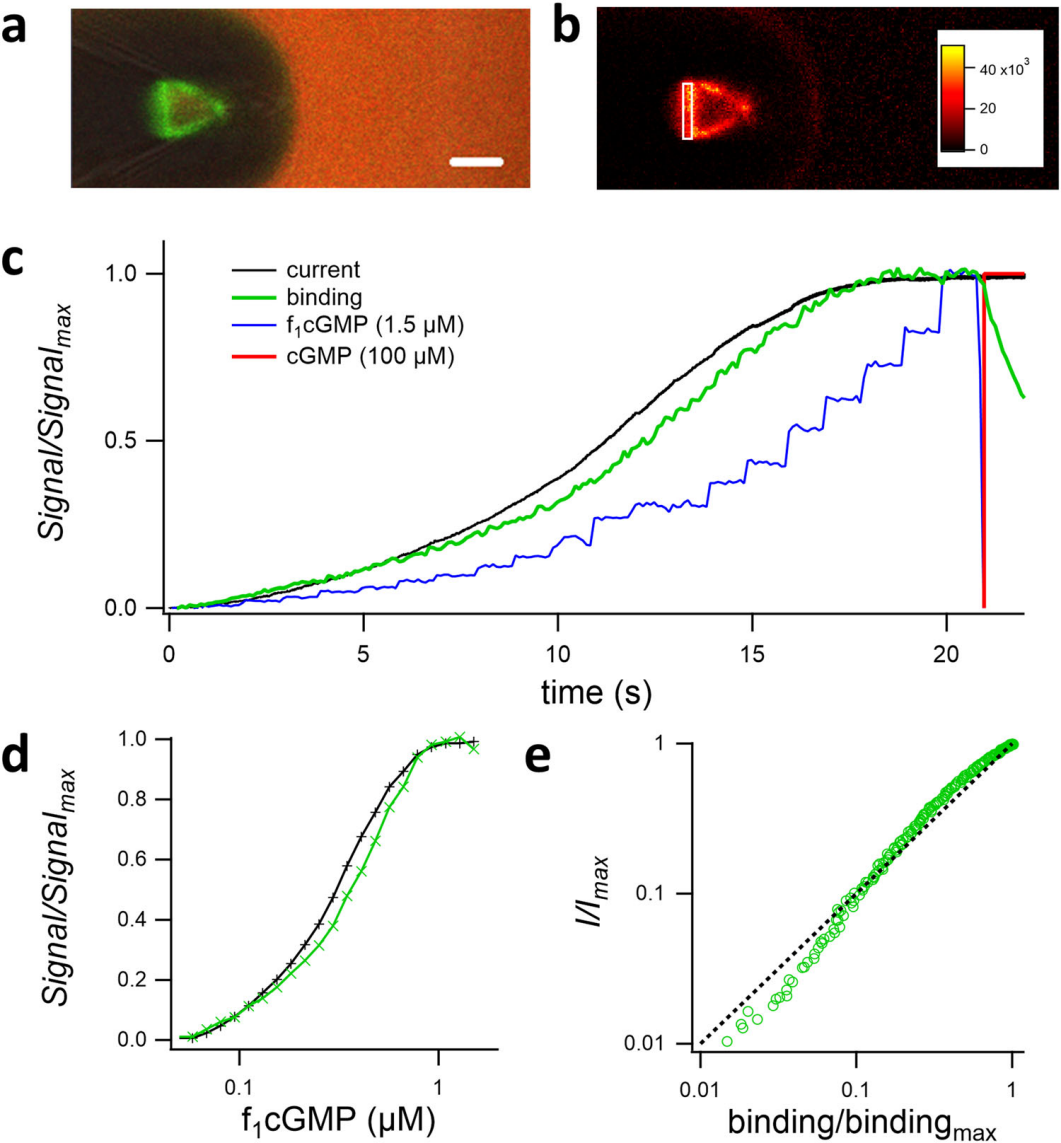
Ligand binding and activation in CNGA2 channels at or close to equilibrium. In inside-out patches of *Xenopus* oocytes containing CNGA2 channels, the channels were activated by cGMP in the bath solution in 23 consecutive steps that were approximately equidistant on a logarithmic scale. The voltage was +50 mV. **a** Micrograph of an inside-out patch with 1.5 µM f_1_cGMP (green) representing both binding signal as well as signal from ligand solution and 4.25 µM Dy647 (red) as counterstaining solution, scale bar indicates 5 µm. **b** Scaled difference image representing the binding signal alone. The average of the patch dome as indicated by the ROI is the binding signal. **c** Concentration steps (blue, from the bulk signal) with 1000 ms duration where applied, the corresponding current (black) and binding signal (green) are shown. f_1_cGMP was replaced by 100 µM cGMP (red). Fluorescence signals are normalized to maximal binding, and current signals to the current from saturating, unlabeled cGMP. The plot also confirms that f_1_cGMP is a full agonist for CNGA2. **d** Current of binding signal and current at the end of a concentration step vs. the applied concentration. Note that the crossover of binding and current amplitudes, indicative of a complex subunit cooperativity. **e** Normalized current signal vs. normalized binding signal. The dashed line indicates a slope of unity. For a low signal, the binding precedes the current amplitude while at a higher binding signal, the influence on the current is reduced.

Our technique enables also to record in parallel measurement of current and ligand binding as an orthogonal signal. To demonstrate this, we performed such measurements with CNGA2 channels (Fig. 4). For monitoring ligand binding, we used confocal patch-clamp fluorometry (cPCF)^1,22^ and the fully efficient and potent fluorescent agonist f_1_cGMP (*8-[Cy3B]-AHT-cGMP*)^23^. To detect the binding of the labeled ligand in the presence of a free labeled ligand in solution, the solution was counterstained by an inert fluorophore (Fig. 4a). Subsequently, a scaled difference image representing the pure binding signal can be calculated (Fig. 4b)^1,22^ and the average of the dome of the patch can be evaluated as a binding signal. As an example of equilibrium, or quasi-equilibrium, conditions, the fluorescent ligand was applied while increasing its concentration in a stepwise fashion. This was followed by a saturating concentration of the unlabeled agonist to confirm that the fluorescent ligand is indeed a full agonist (Fig. 4c). Detailed concentration–binding and concentration–activation relationships can be simultaneously derived (Fig. 4d). Using an ROI near the pipette, one can additionally measure the signal from the ligand in solution which is proportional to the ligand concentration. Detecting an orthogonal signal with the MFD is an experimental challenge because detecting binding within the patch requires high NA images to ensure small voxels which is usually associated with a small working distance. Notably, moving a 3 mm wide MFD directly next to a coverslip and the pipette tip induces considerable mechanical strain on the patch that reduces its stability. It is also notable that the imaging rate is slower than typical electrophysiological sampling rates which also limits the time resolution of the optical part of the cPCF recordings. Nevertheless, the relation of binding and current signal can be directly read out (Fig. 4e). It shows that binding precedes current at low ligand concentrations while at higher concentrations, and thus at a high degree of binding, the current increase per binding is reduced.

### Using the power of the MCSA for studying time-dependent signals

In order to extract kinetic information from current signals following concentration steps, we considered the features of the MCSA to determine rate constants for channel gating.

Before analyzing data, three challenges had to be addressed:

1. It is essential to consider the speed of the solution changes obtained with our system. Being aware that our MFD has thicker walls between the channels (cf. Fig. 1b) as compared to walls in pulled theta glass pipettes optimized for fastest solution exchange^6,8^, our approach is a priori some slower. Therefore, we did not follow the strategy to maximize the speed of the switch by sophisticated technical approaches^9,24^ but determined the real-time course of solution exchange at each individual patch and included this time course in the quantitative analysis of the observed current kinetics. In fact, less than the maximum piezo speed was employed to avoid vibration artefacts.
  To determine the real-time course of solution exchange, we switched in each patch the concentration of the permeating K^+^ ions from 150 to 120 mM at maximally opened channels evoked by a saturating ligand concentration. This leads to an inwardly directed current change. For this purpose, two outlets of the MFD were reserved.
  For excised patches, located in the interior of the pipette, typical time constants were in the range of several milliseconds, or sometimes even more. These transients, arising from a changed K^+^ concentration at the patch, we used to calculate the time course of the ligand concentration at the patch. For these considerations, we assumed a cylindrical geometry between the pipette tip and patch with the length *L* which considerably varied among the patches.
  We then solved the diffusion equation for this cylinder and calculated the concentration time course at the patch by using its specific diffusion coefficient. Thereby, we assumed a virtual second cylinder to describe the molecules that are reflected by the membrane (Supplementary Fig. 3a). The solution of the diffusion equation for this double cylinder is
  1 $${c}\left({L},{t}\right)={{c}}_{2}+\left({{c}}_{1}-{{c}}_{2}\right)\frac{4}{{\pi }}\mathop{\sum }\limits_{{n}=1}^{{\infty }}\frac{-{(-1)}^{{n}}}{(2{n}-1)}\exp \left[-{D}{\left(2{n}-1\right)}^{2}\frac{{{\pi }}^{2}}{{4{L}}^{2}}{t}\right]$$
  where *c*(*L*,*t*) is the concentration of the molecule at time *t* and the membrane position *L*, *c*_1_ the initial concentration, *c*_2_ the final concentration after infinite time, and *D* the diffusion coefficient.
  We then noticed that after an initial sigmoidicity, these time courses approximate a monoexponential function with a delay *t*_0_ (Supplementary Fig. 3b), given by the first summand only (*n* = 1), resulting in
  2 $$\begin{array}{cc}{c}\left({L},{t}\right)\,{\approx }\,{{c}}_{2}+\left({{c}}_{1}-{{c}}_{2}\right)\exp \left[-\frac{1}{{\tau }}({t}-{{t}}_{0})\right] & {{{{{\rm{with}}}}}}\,{{t}}_{0}={\tau }{{{{\mathrm{ln}}}}}\left(\frac{4}{{\pi }}\right)\approx 0.24{\tau }\end{array}$$
  and the time constant ${\tau }=\frac{{4{L}}^{2}}{{{D}{\pi }}^{2}}$.
  For practical reasons, Eq. (2) can be modified to
  3 $${c}\left({L},{t}\right)=\left\{\begin{array}{cc}0 \hfill & {{\rm {for}}\; t} < {{t}}_{0}\\ {{c}}_{2}+\left({{c}}_{1}-{{c}}_{2}\right)\exp \left[-\frac{1}{{\tau }}\left({t}-{{t}}_{0}\right)\right] & \,{{\rm {for}}\; t}{\ge }{{t}}_{0}\end{array}\right.$$
  The derivation of these equations is described in Supplementary Note 1.
  After fitting an exponential to the time course evoked by the K^+^ ion concentration jump, one can calculate the ligand concentration time courses at the patch by using the fitted time constant *τ* and the ratio of published diffusion coefficients of K^+^ ions and the ligand investigated.
2. Because it is a priori not clear whether or not a ligand step is sufficiently long to reach an equilibrium, we dropped for the kinetic analyses any assumption of real equilibria. Instead, we used kinetic models covering both sequential ligand binding and conformational changes in time.
3. We finally had to test the influence of the limited speed of the solution exchange on the conclusiveness of the analyses. To this end, we performed a series of simulations and fits with a C–O model, containing solely a single ligand-depending opening step (Supplementary Note 2, Supplementary Fig. 4a). The results of these analyses can be summarized as follows: (1) With noiseless time courses of the open probability, the rate constants could be determined with high accuracy, even when the time constant of the solution exchange, *τ*_s_, exceeded the activation time constant *τ*_a_ of the C–O model 50 fold (Supplementary Fig. 4b). The resolution is apparently limited only by the accuracy of the computations and assumptions. (2) Even with more realistic noise in the time courses, generated by the stochastic activity of 1000 channels, activation time constants exceeding the solution exchange by 10- or 50-fold, certainly unfavorable assumptions, allowed us to determine the rate constants with reasonable accuracy (Supplementary Fig. 4a, c; Supplementary Table 1). This suggests for our solution steps of several milliseconds that rate constants in the range of hundreds of microseconds can indeed be determined. (3) Erroneous estimates of *τ*_s_ by up to 10% or 20% enable still useful estimates of the rate constants (Supplementary Fig. 5).

### Analyzing activation kinetics of CNGA2 channels by complex concentration protocols

**Fig. 5:**
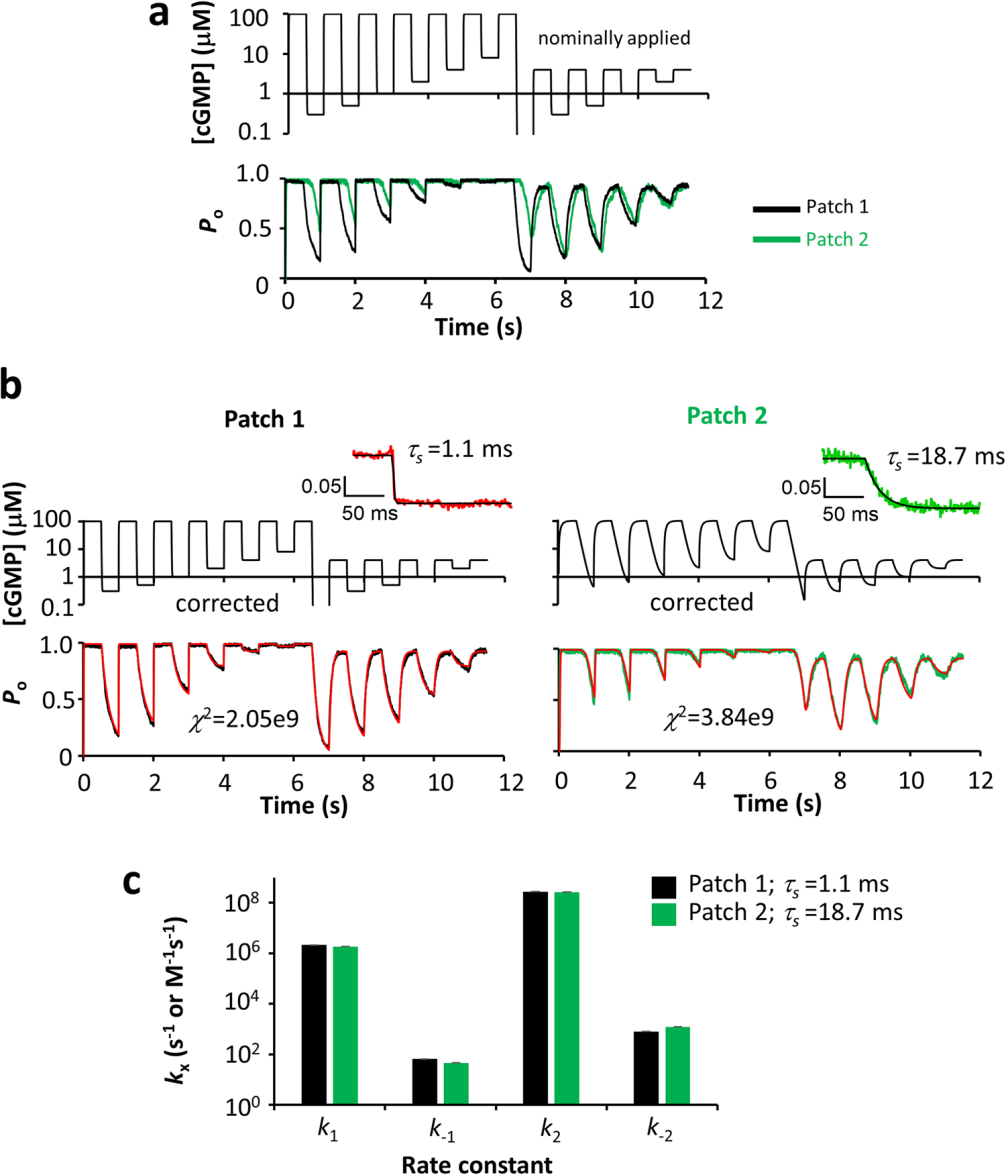
Fit of Model 4*s*4*p* to the data of two patches with notably different speeds of solution exchange. **a** Superimposition of the currents for the patch with *τ*_s_ = 1.1 ms (black) and *τ*_s_ = 18.7 ms (green) following the assumed concentration protocol shown at the top. **b** Effect of correcting the time courses of concentration steps for both patches by using Eq. (3). The insets show the time courses for a K^+^ concentration jump from 150 to 120 mM which were used to fit an exponential function yielding the time constant *τ*_s_. For patch 1, *τ*_s_ was rapid (1.1 ms) whereas for patch 2 *τ*_s_ was exceptionally slow (18.7 ms). While the concentration protocol for patch 1 is close to a perfect rectangular protocol, that for patch 2 is strongly deformed. Both complex current traces were then fitted with the respective corrected concentration protocols using Model 4*s*4*p* (Supplementary Table 2). **c** Rate constants obtained by the fits in (**b**). Despite the highly different complex time courses for the two patches, the corrected pulse protocols yield closely similar rate constants.

To demonstrate the power of our method for kinetic analyses, we analyzed the activation gating of homotetrameric CNGA2 channels by cGMP in terms of sequential kinetic models. The measurements lasted in total only 11.5 s. We applied a complex concentration protocol consisting of 23 cGMP pulses of 500 ms duration as shown in Fig. 5a. The pulses covered the full concentration range between zero cGMP (out of scale in Fig. 5a) and saturating cGMP at 100 μM.

A switch of the K^+^ concentration from −150 to 120 mM in the presence of saturating cGMP was included in the pulse protocol to determine the solution exchange time constant *τ*_s_ at the patch. Based on this *τ*_s_ and the diffusion equation (see Supplementary Note 3), we approximated the actual concentration–time course at the patch (Fig. 5b, top) from the nominally applied pattern of concentration steps (Fig. 5a, top) and applied it in the fit of the currents. The power of this approach is demonstrated by two patches with very different speeds of solution exchange, resulting in very different current traces though the nominal concentration patterns were the same (Fig. 5a, bottom). For patch 1, the solution exchange was rapid (*τ*_s_ = 1.1 ms) whereas for patch 2 the solution exchange was exceptionally slow (*τ*_s_ = 18.7 ms) (insets in Fig. 5b).

We then used Model 4*s*4*p* (Supplementary Table 2), a kinetic model containing four states (4*s*) with two sequential binding steps and one opening step, to fit the complex current responses. The rates for channel opening (e_+_) and closure (e_−_) were set to 990 and 10 s^−1^, respectively, referring to the range found by Li and Lester in single-channel recordings^25^. The four rate constants for binding and unbinding were free parameters (4*p*). The result was that fits using the concentration–time courses corrected by Eq. (3) provided adequate fits (Fig. 5b bottom) with closely similar rate constants (Fig. 5c) despite the markedly different time courses of current and ligand concentrations (Fig. 5a, top, and Fig. 5b top).

For comparison, we tested whether it is possible to use uncorrected, i.e. rectangular, concentration pulses to avoid the described correction protocol, at least for patch 1 with the fast solution exchange. Supplementary Fig. 6 (for fit parameters see Supplementary Table 6) reveals visually that that the fit with rectangular pulses was adequate for patch 1 whereas that for patch 2 failed, a plausible result because the fast solution exchange in patch 1 is closer to a rectangular solution exchange than the slow solution exchange in patch 2. However, using rectangular pulses yielded *k*_*−*1_ and *k*_2_ consistent correlation coefficients of 1 (Supplementary Table 3, top right) which allowed us to determine only their ratio but not their values. In contrast, the corrected time courses provided correlation coefficients larger than −1 and smaller than 1 for all parameters (Supplementary Table 3, top left). This enabled us to determine the values of all free parameters. The same conclusion holds for the slow patch 2 (Supplementary Table 3, bottom). Hence, for our Model 4*s*4*p* the described correction protocol for the concentration time courses yielded a second benefit: determinability of all free parameters. We speculate that this result is due to the increased complexity of the actual time—causes adding additional constraints that are not sufficiently mirrored in the rectangular pulses.

We finally tested on the example of patch 1, whether the complex concentration time course allows for evaluating the required model complexity, in particular, whether cooperativity of the subunits can be detected. To this end, we fitted the time course of patch 1 with kinetic models containing 1–4 binding steps with either the assumption of independent (identical) binding sites or allowing for different—thus cooperative—binding sites. Analog to Model 4*s*4*p*, the models are specified by the number of states (*s*) and the number of parameters (*p*) (Supplementary Table 2). The equilibrium rates for channel opening (e_+_) and closure (e_−_) were again set to 990 and 10 s^−1^.

**Fig. 6:**
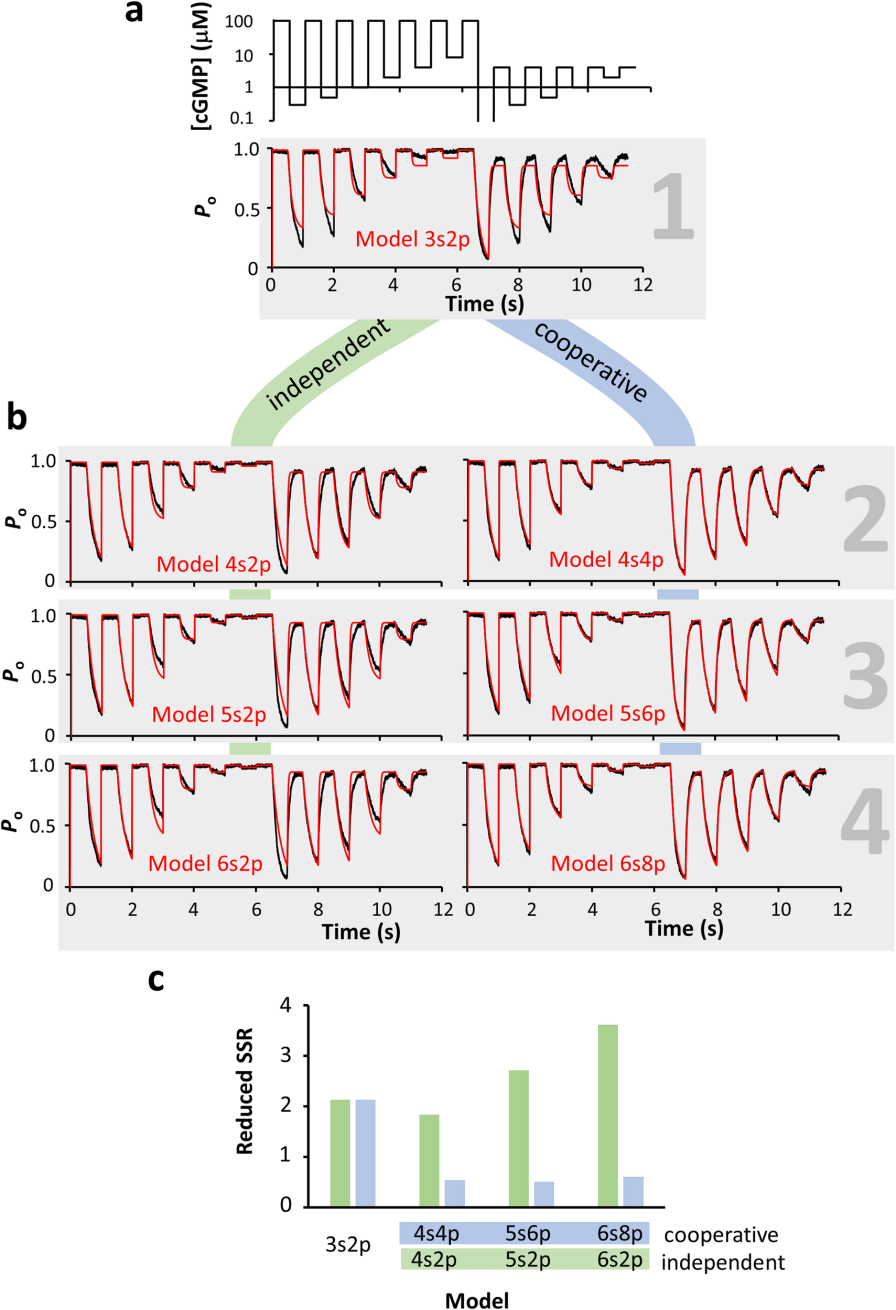
Fit of kinetic schemes to complex data of CNGA2 channels with either independent or cooperative ligand binding. Inside-out macropatch. The time constant for the solution exchange determined by a K^+^ jump was 1.1 ms. The rate constants for channel opening *(e*_*+*_*)* and closure *(e*_*−*_*)* (see Supplementary Table 2) were set to 990 and 10 s^−1^ ^25^. The open probability, *P*_o_, was respectively normalized. **a** Complex protocol of concentration pulses (top) plotted with a logarithmic ordinate. The concentration between 6.5 and 7 s was zero and is therefore not plotted. The whole recording lasted 11.5 s. The sampling rate was 2 kHz. Pulses into 120 mM KCl to characterize the solution exchange are not shown. The corresponding current time course (bottom, black) shows slow activation and deactivation kinetics. The fit (red) with Model 3*s*2*p*, assuming one binding step only (right gray number), does not describe the data well. **b** Continuous model expansion: Comparison of sequential models with two to four binding steps assuming either independent or cooperative binding. The models and the rate constants are provided in Supplementary Tables 2, 4, and 5, respectively. **c** Plot of the reduced summed squared residuals (reduced SSR, see Supplementary Methods) of the fits in (**b**). While independent binding does not improve the fit, a second cooperative binding step causes a significant improvement that is not further improved by a third or fourth binding step.

The fit with Model 3*s*2*p* (red) is only poor (Fig. 6a). Extending the models to two, three, and four binding steps, with either forced independent or potentially cooperative binding (Fig. 6b; Supplementary Table 2), improved the fits (for rate constants see Supplementary Tables 4 and 5). Plotting the respective reduced summed squared residuals (reduced SSR; see the “Methods” section) of the fits yields that independent binding by two, three, or four ligands does not lead to a relevant improvement of the fits (Fig. 6c). In contrast, a second cooperative binding step causes a significant improvement of the fit while binding of a third or fourth ligand is without further effect. Hence, the second binding step plays a key role in channel activation. This result matches our previous results obtained by extensive and time-consuming measurements with cPCF^1,5^. It should be noted that the fits with Model 5*s*6*p* and Model 6*s*8*p* were underdetermined, i.e. did not converge to a minimum (100 iterations). For the future, it is highly attractive to include in such measurements a second orthogonal signal, as ligand binding (cf. for Fig. 4c), to gain further insights into the activation process.

## Discussion

Even after intensive work over many decades, the phenomenon of cooperativity of subunits in oligomeric ligand-gated ion channels is still elusive. Herein we describe a methodological package, to gain within a couple of seconds complex functional information about subunit cooperativity, and we demonstrate the potency of the method for trimeric P2X2 and tetrameric CNGA2 channels.

The central elements of our MCSA are a chip-based microfluidic device (MFD), containing 30 channels for different solutions, and a control unit driven by a computer. The approach combines four key features: (1) Sequences of a large number of concentration steps with freely designable duration can be applied. This enables us to determine either concentration–activation or concentration–binding relationships in a few seconds at or near equilibrium or both. (2) In addition to staircase-like changes of the ligand concentration, also freely designable highly complex concentration protocols can be applied. (3) The solution steps are sufficiently rapid to quantify time courses of activation and deactivation as well ligand binding and unbinding if using fluorescent ligands and confocal patch-clamp fluorometry. (4) Diffusion time courses of the ligand to the receptors can be included in the analysis for correction of time-dependent current signals, which allows the investigator to interpret the signals by kinetic schemes, thereby surrendering the assumption of any equilibria.

Notably, the rapidity of the solution application minimizes the risk of any run-down processes and other disturbing changes in the patch stability. This allows to repeat of highly complex sequences of solution pulses after a couple or tens of seconds, and thus reproduces the data in the same excised membrane patch or whole cell. An additional advantage of the MFD is the minute reagent consumption.

We determined for inside-out patches located within the pipette the actual speed of the solution steps by varying the K^+^ concentration at fully activated channels. We quantitatively described this process and included it in the mathematical interpretation of the results. To this end, we used the diffusion equation and the diffusion coefficient of the ligand to calculate the actual time course for the change of the ligand concentration at the patch. For excised patches in the interior of the pipette, we showed for two patches with highly different speeds of the solution exchange that the diffusion along the distance between the pipette tip and the patch is a relevant determinant for the measured kinetics of receptor activation (Fig. 5). Quantitative modeling of this diffusional process led to similar rate constants for receptor activation in the two patches, while ignoring this diffusional difference did not. Practically, we determined the time courses for solution exchange at the membrane by stepping the K^+^ concentration at the internal face of the channels from 150 to 120 mM. Fitting this time course with Eq. (3), derived from the diffusion equation and knowledge of the diffusion coefficients for K^+^ ions, *D*_K_ = 1.96 × 10^−5^ cm^2^ s^−1^ ^26^ (p. 268) allows also to estimate the patch position *L*. Knowing the distance *L* allowed us to determine the time course for the solution exchange with cGMP by using the diffusion coefficient for the structurally closely similar cAMP, *D*_cAMP_ = 4.4 × 10^−6^ cm^2^ s^−1^ for cAMP^27^. The process of convection outside the pipette due to stepping the solution from one channel to the next was apparently without major influence.

Though the rise times of our solution exchange were not as fast as reachable by theta-glass pipettes, they are not limiting for measuring concentration–activation (Figs. 2, 3a,b) and concentration–binding relationships at or near equilibrium (Fig. 4c). For slowly gating channels, they are certainly also sufficient for kinetic analyses. Beyond this we showed both theoretically and experimentally that it is a priori not necessary to realize extremely fast solution changes if only the kinetics of the solution changes at the patch can be determined as performed herein by the steps of the K^+^ concentration. Using a C–O model, we also showed by simulations that with time courses even 50 times slower than the relaxation time constant, a reasonable estimate for the rate constants can be obtained in the presence of a realistic noise level. This enabled us to work with a relatively low flow speed of the test solutions of ~70 mm s^−1^, which ensures increased patch stability and thus more and longer measurements. Solution switches with moderate speed provide the advantage of better mechanical stability for whole cells positioned at the outside of the pipette tip. Our approach to determine the real-time course of the solution exchange and to include it in the interpretation of the data can be adapted to whole-cell recordings as well.

Solution switches with theta glass, or related capillaries, are widely used. As pointed out above, the thinner walls in the application pipettes of theta glass can a priori produce faster solution switches than performed herein^6,7,9,10,28^. Also, sophisticated high-speed techniques, using special circuits compensating for mechanical interferences, have been reported, changing between two solutions within tens of microseconds^24^. Though very elegant, all these approaches have to face the diffusional limitations within the patch pipette, limiting the time resolution. In contrast to our approach, a theta glass pipette cannot apply sufficiently many solutions to record complete concentration–activation or concentration–binding relationships in a couple of seconds, in particular, if these relationships contain multiple components. Another classical approach is using manifolds or *Y*-connectors^29,30^, which require, however, a higher reagent consumption and longer experimental times due to the exchange of the dead volume. Thus, the strength of our experimental approach is the combination of many immediately available solutions with a sufficient rapidity of the concentration steps combined with determining the time course of the concentration change.

Multiple parallel solution streams for patch-clamp analyses have been realized previously by a microfluidics-patch clamp platform designed for high throughput screening. As herein the microfluidic chip contains multiple channels arranged in parallel and the chip is mounted on the motorized scanning stage which forms the experimental chamber^31,32^. The walls between the fluid channels in this device are 22 μm thick (versus ~12 μm herein) and the whole device including the whole bath solution is moved linearly to exchange the solutions whereas we enhanced the solution switch by including a piezo actuator. We estimate from their recordings that the speed of the solution exchange is an order, or even some more, slower than the speed reached by our system. The authors could determine concentration-activations at equilibrium but, due to the much slower speed, more detailed kinetic analyses were not reported. To some extent, similarity exists to an also free-standing microfluidic pipette made by poly(dimethylsiloxane) with a circulating liquid tip generating a self-confining volume in front of the outlet^33^. This highly sophisticated pipette is able to carry out various complex operations, such as mixing, multiplexing, or gradient generation at selected cells in cell cultures. However, the solution exchange by this technique is also more than an order of magnitude slower and, moreover, it also cannot administer multiple solutions in a few seconds as performed herein.

Our method has great potency also for other ligand-gated ion channels because the readout of currents is straightforward. In particular, concentration–activation relationships with more than 20 data points at or near equilibrium can be obtained within seconds, allowing thus to easily identify different components. The numerous outlets also allow the investigator to perform kinetic studies with combinations of agonists, allosteric effectors, and antagonists.

In combination with the parallel recording of concentration-binding relationships at or near equilibrium by cPCF^1,22^ and fluorescent ligands (Fig. 4c), our method can provide new valuable information on the activation gating of these channels if only a fluorescent ligand with sufficient affinity is available. In general, combining orthogonal signals, e.g. current and binding as done in cPCF, increases the identifiability of model parameters considerably, e.g. to unhide classes of states in hidden Markov models. Newer algorithms^34^, taking the particular noise signature into account, will further improve modeling quality.

The temporal resolution of such measurements is limited by the speed of the confocal microscope. For cPCF measurements, further work is needed to include the solution exchange, because calculating difference images so far does not account for concentration gradients within the pipette. Strategies using alternating reference dye concentrations might solve this current limitation. Nevertheless, the simultaneous measurement of binding and gating allows to detect the relation between ligand binding and channel activation within several seconds (Fig. 4e) without requiring information on the actual ligand concentration at the patch.

Beyond this, it is feasible to adapt our method to metabotropic receptors if the conformational changes can be read out properly, e.g. by fluorescence-based techniques^35–38^. In those cases, it would be possible to determine the speed of the solution exchange by co-expressing an appropriate ion channel or recording leak currents^39^.

Concerning the shown experiments with complex patterns of concentration pulses (Figs. 5,6), there is a similarity of our analysis to a previous strategy using wavelet-based protocols to analyze the gating of voltage-sensitive Shaker channels^40^. In these analyses, the author specified a fluctuating voltage input and applied it as command voltage in the patch-clamp. As a result, the author was able to discriminate between different gating models which was not possible with current signals arising from a series of voltage-clamp pulses typically used in electrophysiology. If following these ideas, it is imaginable for the future to develop more specifically tailored concentration protocols than those used herein to optimize model discrimination and to develop these strategies in the direction of recording orthogonal signals by cPCF, of either ligand binding and activation gating, ligand binding and conformational changes, or conformational changes and activation gating.

## Methods

### Fabrication of the microfluidic solution device (MFD)

The preparation of the chip device for the multichannel-flow system started with a (110)-Si wafer (p-type; diameter 100 mm; thickness *d* = 525 µm), which was covered by a thermal oxide (thickness 1 µm, both sides) and a 50 nm NiCr (80:20) hard mask (upper side). The micro-channels were defined by standard photolithography (3 chips on the wafer). After development, the mask layers are opened by means of wet etching. The actual microfluidic channels were etched using reactive ion etching with an inductively coupled plasma (ICP) with a combination of CH3, SF6, and O_2_ gases. When the desired depth was reached, the mask layers were removed. The wafer was then sealed with a glass wafer by anodic bonding and diced into three independent chips. The outlet of the chips was sealed with wax for protection and the glass wafer was cut, sloped, and polished with diamond grinding paste. This sloping was required to be able to move the outlets close to the bottom of the experimental chamber. After cleaning and thermal water removal, two-component silicon was used to cast an interface to the tubings coming off the valve assembly that controls the flow from 30 1-mL syringes.

### Quantification and correction of ligand diffusion between pipette tip and patch

To determine the real-time course of solution exchange, we switched in each patch the concentration of the permeating K^+^ ions from 150 to 120 mM at maximally opened channels evoked by a saturating ligand concentration. These data were fitted by Eq. (3), and solution exchange of the ligand was estimated from this using Eq. (2). See Supplementary Note 1 for derivation and Supplementary Note 3 for a discussion of these equations.

### Molecular biology and channel expression

Stable HEK293 cells containing a rat-P2X2 channel construct (His-rP2x2-StrepII, Accession number: NM_053656) were induced with tetracyclin using the T-Rex system (Invitrogen) and used 24–48 h after transfection. The cell line was kindly provided by F. Markwardt, Halle.

Rat CNGA2 channels (Accession number: AF126808), were transiently expressed in oocytes from adult females of *Xenopus laevis*. The oocytes were obtained surgically under anesthesia (0.3% 3-aminobenzoic acid ethyl ester). The procedures had approval from the authorized animal ethical committee of the Friedrich-Schiller University Jena (Germany). The oocytes were incubated with collagenase A (3 mg/ml, Roche, Grenzach-Wyhlen, Germany) for 105 min in Ca^2+^-free Barth´s solution containing (in mM) 82.5 NaCl, 2 KCl, 1 MgCl_2_, 5 HEPES, pH 7.5. After this incubation, the oocytes of stages IV and V were manually dissected and injected with ~50 ng of cRNA encoding CNGA2 channels. After injection with cRNA, the oocytes were cultured at 18 °C for 3–4 days in Barth medium containing (in mM) 84 NaCl, 1 KCl, 2.4 NaHCO_3_, 0.82 MgSO_4_, 0.41 CaCl_2_, 0.33 Ca(NO_3_)_2_, 7.5 Tris, pH 7.4. Part of the media contained in addition to Cefuroxim, Penicillin/Streptomycin.

### Electrophysiology

Macroscopic currents were recorded with the patch-clamp technique^41^ from either whole HEK293 cells, or outside-out patches thereof, and from inside-out patches of *Xenopus laevis* oocytes. The patch pipettes were either pulled from borosilicate glass tubing (outer and inner diameter of 2.0 and 1.0 mm, respectively; Hilgenberg GmbH, Malsfeld, Germany) or quartz tubing (outer and inner diameter of 1.0 and 0.7 mm, respectively; VitroCom, New Jersey, USA). cPCF experiments were performed with borosilicate glass tubing. The pipette resistance was 1 to 1.7 MΩ. The bath and pipette solution for CNGA2 measurements contained (in mM): 150 KCl, 1 EGTA, and 10 Hepes (pH 7.4 with KOH). For varying the concentration of the permeating ions, KCl in the bath solution was lowered to 120 mM. Measuring solutions for P2X2: The pipettes were filled with intracellular solution containing (in mM) 142 NaCl, 5 Bapta, 5 EGTA, and 10 HEPES, pH 7.4. The pipette resistance was 2.5–6.0 MΩ. The bath solution contained (in mM) 142 NaCl, 10 EGTA, 10 HEPES, and 10 Glucose, pH 7.4.

Electrophysiology was performed with an Axiopatch 200B (Axon Instruments, Foster City, CA) amplifier controlled either by the ISO3 hard- and software (MFK, Niedernhausen, Germany) or by a LIH8-8 data acquisition interface and patchmaster software (HEKA GmbH, Lambrecht, Germany). The sampling rate for the current recording was 10 kHz (whole cell) or 2 kHz (outside out) for P2X2 channels and 1 to 10 kHz for CNGA2 channels increasing with decreasing step-times: 1000 data points were sampled per concentration step. The filter (4-pole Bessel) was set to half the sampling rate, but minimally to 1 kHz.

### Statistical analysis and data evaluation

The amplitude of late currents was determined as the mean current over the last 0.6 ms of the pulses for P2X2 and the last 5% of step duration for CNGA2 measurements. Data are given as mean ± s.e.m. If not otherwise noted, concentration-activation relationships at or close to the equilibrium were fitted with IgorPro 7.0.8.1 (WaveMetrics, Lake Oswego, OR, USA) by

4 $$I/I_{\rm {max}} =1/(1+({\rm {{EC}}}_{50}/[{{\rm {cGMP}}}])^{n}).$$

*I* is the actual current amplitude, and *I*_max_ the maximum current amplitude at the saturating ligand concentration specified for each patch. EC_50_ is the ligand concentration generating the half maximum current and *n* the Hill coefficient. Zero and saturating concentrations were not included in the fit as they were used in offset correction and normalization, respectively.

### Global fit of kinetic models

For fitting complex concentration patterns (Fig. 5, Supplementary Table 2) with limited speed of the solution exchange, we determined the time courses of the solution exchange as described in the main manuscript by a monoexponential fit (see Fig. 6b) and generated the concentration profiles with the determined time constant. The step width (sampling interval) of the concentration changes was 0.5 ms. The complex pulse protocols were used as command pulses.

The global fit strategy was performed with the MATLAB software described in an earlier report of our lab to analyze rate constants in HCN2 channels and P2X2 receptors^42^.

In brief:

For fitting the Markov models, the equation

5 $$\frac{{{{{{\rm{d}}}}}}{{{{{\bf{p}}}}}}({t})}{{{{{{\rm{d}}}}}}t}={{{{{\bf{p}}}}}}\left({t}\right) \cdot {{{{{\bf{Q}}}}}}({L},{{{{{\rm{\theta }}}}}})$$

was solved with the Eigenvalue method. **p**(*t*) is the row vector of the probabilities to be in one of the model states and **Q**(*L*,*θ*) is the **Q**-matrix, depending on the concentration *L* and the vector **θ** of the model parameters *θ*_*j*_. To obtain the open probability *P*_o_ of the channel, the components of **p**(*t*) from the open states are included.

Varying the model parameters step by step, using a modified Levenberg–Marquardt algorithm, the program finds an optimal set of parameters of **θ** for a minimum difference between the experimental and the calculated curves. The criterion for the best fit was the minimized summed *χ*^2^ over the number *n* of the given time points *t*_*i*_ according to

6 $${{\chi }}^{2}=\mathop{\sum }\limits_{1=1}^{{n}}\frac{{[{{P}}_{{{{{{\rm{o}}}}}},{{{{{\rm{m}}}}}}}\left({{t}}_{{i}}\right)-{{P}}_{{{{{{\rm{o}}}}}},{{{{{\rm{c}}}}}}}({{t}}_{{i}},{{{{{\boldsymbol{\theta }}}}}})]}^{2}}{{{\sigma }}^{2}({{t}}_{{i}})}=\frac{1}{{\bar{{\sigma }}}^{2}}\mathop{\sum }\limits_{{i}=1}^{{n}}{[{{P}}_{{{{{{\rm{o}}}}}},{{{{{\rm{m}}}}}}}\left({{t}}_{{i}}\right)-{{P}}_{{{{{{\rm{o}}}}}},{{{{{\rm{c}}}}}}}({{t}}_{{i}},{{{{{\boldsymbol{\theta }}}}}})]}^{2}=\frac{{S}}{{\bar{{\sigma }}}^{2}}$$

The squared deviations between the measured and calculated open probabilities *P*_o,m_(*t*_*i*_) and *P*_o,c_(*t*_*i*_), respectively, were weighted by their reciprocal variance. Assuming that all variances are about the same, ${{\sigma }}^{2}\left({{t}}_{{i}}\right)\approx {\bar{{\sigma }}}^{2}\forall {{t}}_{{i}}$, only the sum *S* of the squared differences has to be minimized.

The mean variance ${\bar{{\sigma }}}^{2}$ can be estimated to

7 $${\bar{{\sigma }}}^{2}=\frac{{S}}{{n}-{k}-1}$$

where *k* is the number of fitted parameters.

Inserting ${\bar{{\sigma }}}^{2}$ into Eq. (6) yields the minimum summed *χ*^2^ as well. The Levenberg-Marquardt algorithm now provides the covariance matrix, providing by the main diagonal elements (cov)_*jj*_ the standard errors SD(*θ*_*j*_) of each parameter *θ*_*j*_ according to

8 $${{{{{\rm{SD}}}}}}\left({{{{{{\rm{\theta }}}}}}}_{{j}}\right)=\sqrt{{({{{{\mathrm{cov}}}}})}_{{jj}}}$$

### Correlation matrices

To compute the correlation matrix for the parameters of Model 4*s*4*p*, we used the respective covariance matrix cov_*kx*_ as provided by the fit and calculated the elements *ρ*_*i*,*j*_ of the correlation matrix, corr_*kx*_, according to

9 $${{\rho}}_{{ij}}={{{{{\mathrm{cov}}}}}}_{{kx}}({i},{j})/({{\sigma }}_{{i}}{{{\cdot }}{\sigma }}_{{j}}).$$

### Ranking of models and reduced SSR

For each model the summed squared residuals (SSR) were calculated and, additionally, the number of parameters needs to be accounted for. This is done by normalizing the SSR on the degrees of freedom minus the number of free parameters, resulting in the reduced SSR. Usually, the number of data points is treated as degrees of freedom. Because subsequent data points in relaxation time courses are known to be correlated^34,43^, we opted for a more cautious approach: We count each concentration jump (*n* = 23) as the degree of freedom. This reduced SSR was used to compare models (see Fig. 6c).

### Reporting summary

Further information on research design is available in the Nature Portfolio Reporting Summary linked to this article.

### Supplementary information

Supplementary Information-New

Description of Additional Supplementary Files

Supplementary Data 1

Reporting Summary

## Data Availability

The source data behind the graphs in the paper are available in Supplementary Data 1 and any remaining information can be obtained from the corresponding author upon reasonable request. Software and construction concepts of the MCSA are shared upon reasonable request.
