# Supplementary material for: Fast functional mapping of ligand-gated ion channels
Source: Commun Biol. 2023 Oct 2;6:1003. doi: 10.1038/s42003-023-05340-w (PMC10545696; doi:10.1038/s42003-023-05340-w)
Supplement: Supplementary file 1 — Supplementary Information-New [file 42003_2023_5340_MOESM1_ESM.pdf]

Supplementary Materials to

Fast functional mapping of ligand-gated ion channels

Ralf Schmauder[1], Thomas Eick[1], Eckhard Schulz[2], Günther Sammler[3], Elmar Voigt[4], Günter Mayer[4], Holger Ginter[3], Günter Ditze[3] & Klaus Benndorf[1]

[1] Institut für Physiologie II, Universitätsklinikum Jena, Friedrich-Schiller-Universität Jena, 07743 Jena, Germany

[2] Hochschule Schmalkalden, Fakultät Elektrotechnik, Blechhammer, 98574 Schmalkalden, Germany

[3] Zentrale Forschungswerkstätten, Universitätsklinikum Jena, Friedrich-Schiller-Universität Jena, 07743 Jena, Germany

[4] Leibniz Institut für Photonische Technologien e.V., Albert-Einstein-Straße 9, 07745 Jena, Germany

*Corresponding Authors: K.B. Klaus.Benndorf@med.uni-jena.de or R.S. Ralf.Schmauder@med.uni-jena.de

This PDF file includes:
Supplementary Figures 1-7
Supplementary Tables 1-6
Supplementary Notes 1-3

Supplementary Figures

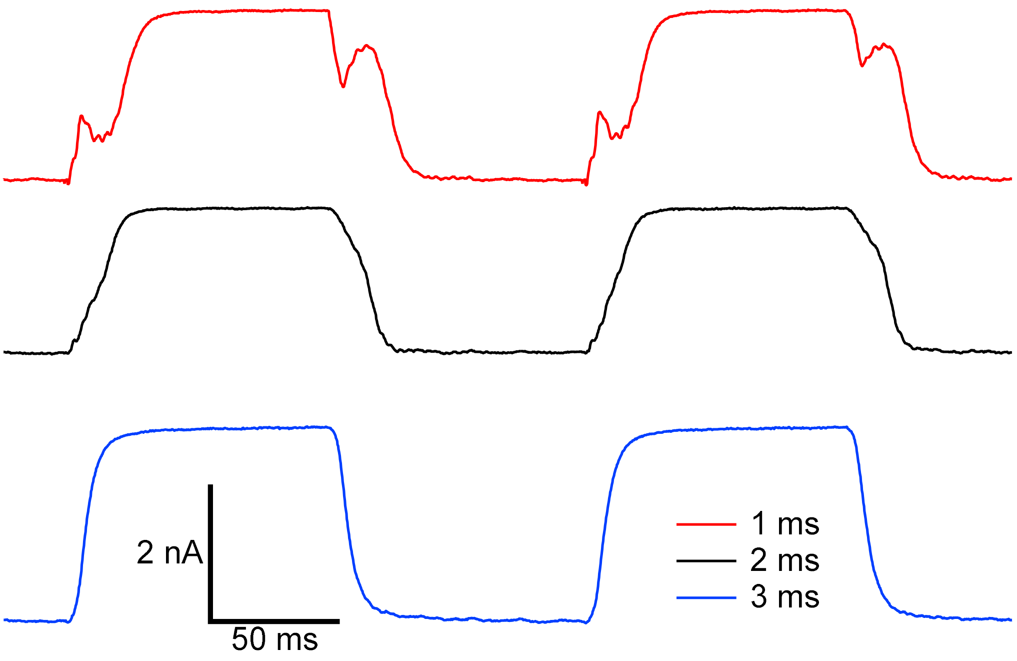

Supplementary Fig. 1 | Reduction of vibrational artefacts by lowering the speed of the piezo. Some vibration artefacts were observed for fast concentration jumps at large borosilicate pipettes (~ 1.5 M Ω). This was most severe for open pipette currents with jumps between 150 and 120 mM KCl at a time-constant of the piezo of 1 ms (top). Reducing the time-constant of the piezo to 3 ms (bottom) reduced the artefacts sufficiently to become neglectable. Note that the time-constant counts for the full 90 µm travel between positions. The kinetics here depend on the position of the liquid junction position and do not represent solution exchange at the pipette tip.

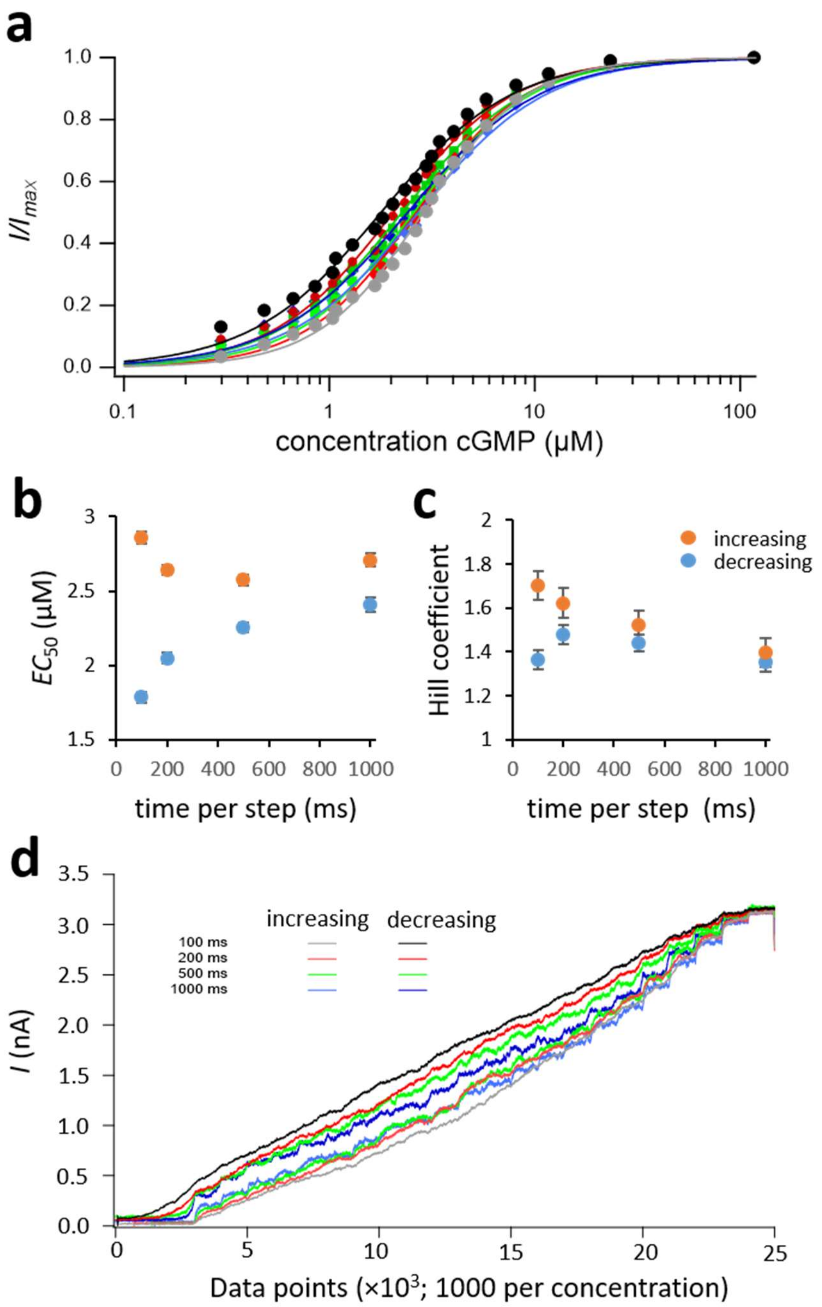

Supplementary Fig. 2 │ Fits of the concentration-activation curves in Fig. 2. a, Fits using the Hill equation. The current amplitude at zero and saturating concentration were set to zero and unity, respectively. These points were not included in the fits. **b,** EC_{50} values of the fits in a as function of the application time. **c**, *Hill* coefficients of the fits in a as function of the application time. **d**, Plot of the data in Fig. 3a against the data points. Note that the hysteresis between increasing and decreasing concentrations varies with both step-length (i.e. time) and concentration. This shows that it is not the settling of the concentration but channel dynamics that is responsible for the hysteresis (see e.g. the lack of hysteresis for 1000 ms pulses at high concentrations).

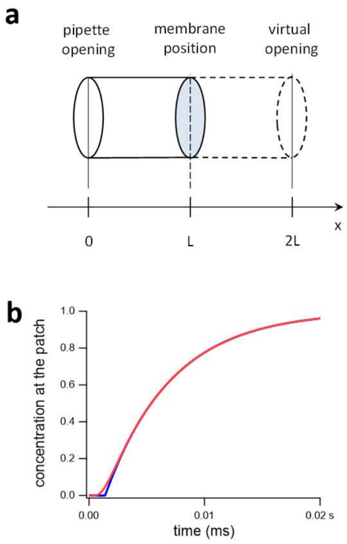

Supplementary Fig. 3 | Quantifying diffusion in the pipette tip. a, Scheme of the mirrored pipette tip used for the computations. For explanation see text. **b**, Simulated diffusion time course (blue) for a normalized concentration jump of the concentration from $c_1 = 0$ to $c_2 = 100\%$ with $D_K = 1.76\times10^{-5} cm^2s^{-1}$ and an assumed $L=5.0\times10^{-6}m$ using equation (1), resulting in $\tau_s = 5.76$ ms and a delay time . The solution time course was simulated with equation (1) with $N_{max}=1000$ (red). A plot obtained with equation (3) using the parameters is overlaid (blue). Fitting with equation (3) results in a systematic error of only less than 2% (not shown).

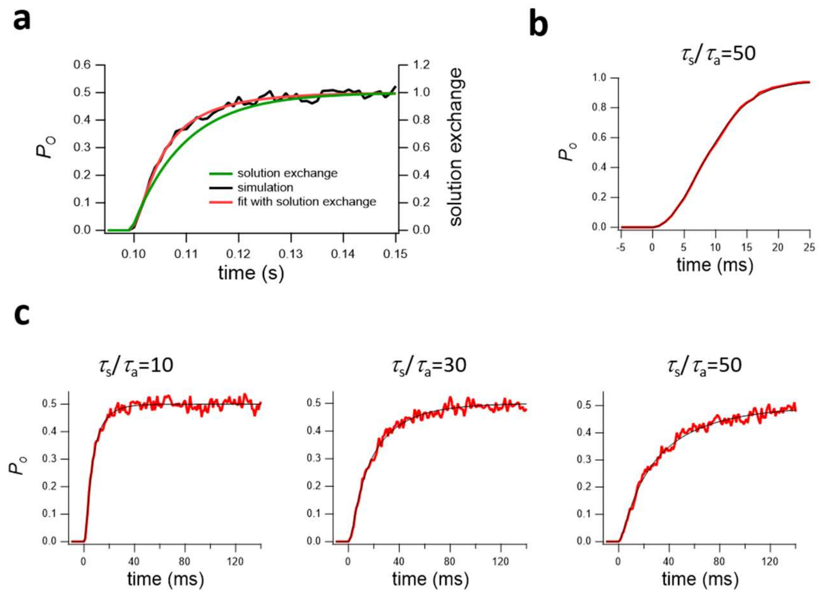

Supplementary Fig. 4 | Simulated time courses of activation in a C-O model. The model is provided by Supplementary Scheme 1 and described in the text. Activation is induced by increasing the ligand concentration L with the time constant of the solution exchange, τ_s. **a,** time courses faster than the solution exchange can be well fitted if the time course of solution exchange is included in the modeling. **b**, Simulated time course of activation in the C-O model k_1 and k_2 where chosen to result in τ_a =1 ms. With P_o=0.99. τ_s/τ_a was set to 50. **c,** Simulated example traces at τ_s/τ_a =10, 30 and 50. P_o at equilibrium was set to 0.5. The noise of the traces arises from the stochastic activity of the channels. Fits were performed as described in Supplementary Methods.

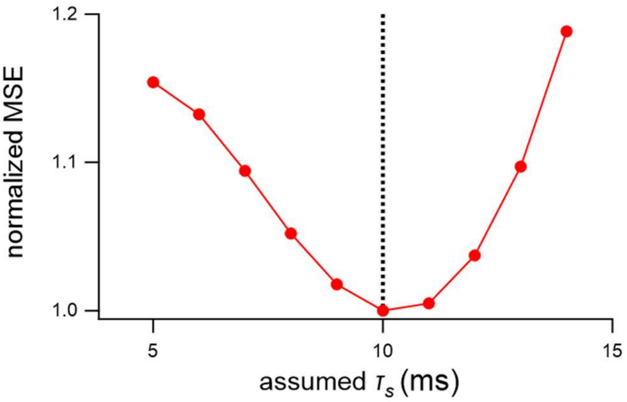

Supplementary Fig. 5 | Accuracy of fit parameters in the C-O model at known speed of concentration change. Data were simulated with the C-O model with an equilibrium P_o of 0.5 and τ_s/τ_a=10 as in Supplementary Fig. 4c (τ_s = 10 ms). Fits were performed with a given variable τ_s. χ^2 (left) showed a clear minimum near the true τ_s.

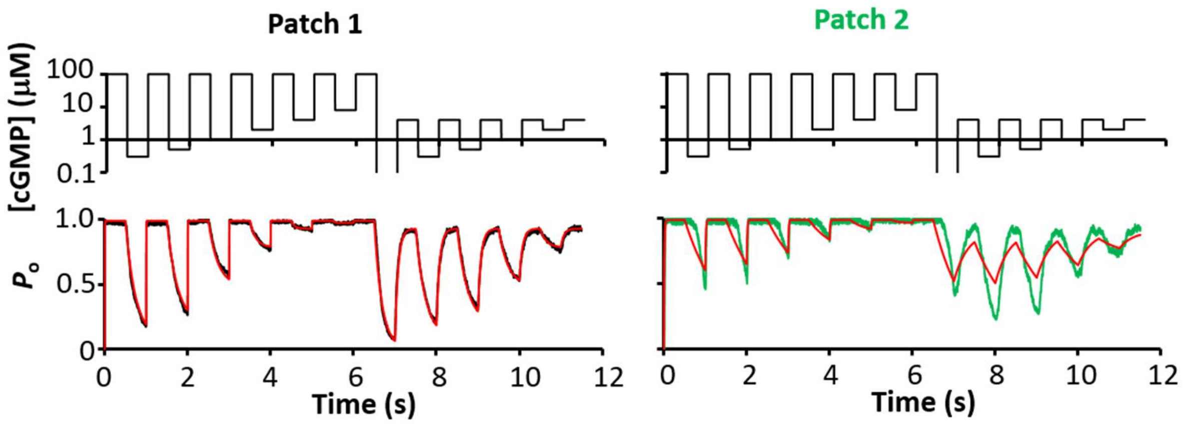

Supplementary Fig. 6 | Fit of Model 4s4p to the data of patch 1 and 2 with rectangular concentration pulses. Visually, patch 1 with fast solution exchange could be fitted reasonably with rectangular pulses whereas the fit for patch 2 with slow solution exchange failed. The fit parameters are provided by Supplementary Table 6. For further explanation see text.

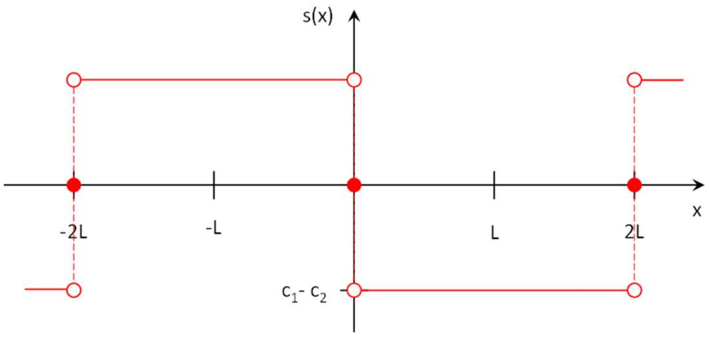

Supplementary Fig. 7 | Concentration profile s(x) used for the computations assuming $c_1<c_2$. For explanation see Supplementary Methods.

Supplementary Tables

		given				fitted					
τ_s (ms)	τ_a (ms)	K_1 ($M^{-1}s^{-1}$)	K_2 (s^{-1})	L (M)	P_o	K_1 ($M^{-1}s^{-}$)	SD (%)	ΔSD (%)	K_2 (s^{-1})	SD (%)	ΔSD (%)
**10**	1	5×10^8	5×10^2	10^{-6}	0.5	4.910×10^8	7.9	7.7	4.912×10^2	7.8	7.6
**30**	1	5×10^8	5×10^2	10^{-6}	0.5	4.705×10^8	9.8	9.2	4.704×10^2	9.9	9.3
**50**	1	5×10^8	5×10^2	10^{-6}	0.5	5.492×10^8	23.4	25.8	5.494×10^2	23.9	26.2
**10**	1	9.9×10^8	10	10^{-6}	0.99	9.935×10^8	2.7	2.7	1.006×10	4.3	4.3
**30**	1	9.9×10^8	10	10^{-6}	0.99	9.981×10^8	3.5	3.5	0.986×10	4.4	4.4
**50**	1	9.9×10^8	10	10^{-6}	0.99	9.879×10^8	3.6	3.6	0.987×10	4.4	4.4

Supplementary Table 1 | Accuracy of rate constants in the C-O model for simulated noisy traces. The traces were obtained with Supplementary Scheme 1 assuming 1,000 channels and using three speeds of solution exchange (τ_s) and an open probability (P_o) of either 0.5 or 0.99. L is the ligand concentration. For further explanation see text.

Model name	Model structure	No. of ligands	Type of ligand binding
3s2p	$C_0 \overset{K_{A1}}{\overset{xk_1}{\underset{k_{-1}}{\rightleftharpoons}}} C_1 \overset{E}{\overset{e_1}{\underset{e_{-1}}{\rightleftharpoons}}} O_2$	1	reference
4s2p	$C_0 \overset{2K_{A1}}{\overset{x2k_1}{\underset{k_{-1}}{\rightleftharpoons}}} C_1 \overset{K_{A1}/2}{\overset{xk_1}{\underset{2k_{-1}}{\rightleftharpoons}}} C_2 \overset{E}{\overset{e_1}{\underset{e_{-1}}{\rightleftharpoons}}} O_3$	2	indepndent
5s2p	$C_0 \overset{3K_{A1}}{\overset{x3k_1}{\underset{k_{-1}}{\rightleftharpoons}}} C_1 \overset{K_{A1}}{\overset{x2k_1}{\underset{2k_{-1}}{\rightleftharpoons}}} C_2 \overset{K_{A1}/3}{\overset{xk_1}{\underset{3k_{-1}}{\rightleftharpoons}}} C_3 \overset{E}{\overset{e_1}{\underset{e_{-1}}{\rightleftharpoons}}} O_4$	3	
6s2p	$C_0 \overset{3K_{A1}}{\overset{x4k_1}{\underset{k_{-1}}{\rightleftharpoons}}} C_1 \overset{3K_{A1}/2}{\overset{x3k_1}{\underset{2k_{-1}}{\rightleftharpoons}}} C_2 \overset{2K_{A1}/3}{\overset{x2k_1}{\underset{3k_{-1}}{\rightleftharpoons}}} C_3 \overset{K_{A1}/4}{\overset{xk_1}{\underset{4k_{-1}}{\rightleftharpoons}}} C_4 \overset{E}{\overset{e_1}{\underset{e_{-1}}{\rightleftharpoons}}} O_5$	4	
4s4p	$C_0 \overset{2K_{A1}}{\overset{x2k_1}{\underset{k_{-1}}{\rightleftharpoons}}} C_1 \overset{K_{A2}/2}{\overset{xk_2}{\underset{2k_{-2}}{\rightleftharpoons}}} C_2 \overset{E}{\overset{e_1}{\underset{e_{-1}}{\rightleftharpoons}}} O_3$	2	cooperative
5s6p	$C_0 \overset{3K_{A1}}{\overset{x3k_1}{\underset{k_{-1}}{\rightleftharpoons}}} C_1 \overset{K_{A2}}{\overset{x2k_2}{\underset{2k_{-2}}{\rightleftharpoons}}} C_2 \overset{K_{A3}/3}{\overset{xk_3}{\underset{3k_{-3}}{\rightleftharpoons}}} C_3 \overset{E}{\overset{e_1}{\underset{e_{-1}}{\rightleftharpoons}}} O_4$	3	
6s8p	$C_0 \overset{4K_{A1}}{\overset{x4k_1}{\underset{k_{-1}}{\rightleftharpoons}}} C_1 \overset{3K_{A2}/2}{\overset{x3k_2}{\underset{2k_{-2}}{\rightleftharpoons}}} C_2 \overset{2K_{A3}/3}{\overset{x2k_3}{\underset{3k_{-3}}{\rightleftharpoons}}} C_3 \overset{K_{A4}/4}{\overset{xk_4}{\underset{4k_{-4}}{\rightleftharpoons}}} C_4 \overset{E}{\overset{e_1}{\underset{e_{-1}}{\rightleftharpoons}}} O_5$	4	

Supplementary Table 2 | Kinetic models used to fit current responses to complex concentration protocols. The models are specified by the number of states (s) including 2-5 closed states and 1 open state, and the number of parameters (p). Stoichiometric factors are assumed. In models with more than one binding step, it is distinguished between independent and cooperative binding. For the case of independent binding, the rate constants of the steps are identical, resulting in only 2 free parameters. In case of cooperative binding, the rate constants of the steps can adopt different values, resulting in 2 through 8 parameters.

The open-closed transition was assumed to be fast. Consistently, the opening rate, e_+, was set to 990 s^{-1} and the closing rate, e_-, to 10 s^{-1}, resulting in a mean open time at saturating cGMP of 100 ms. It is a remarkable result that our approach enables to observe activation kinetics much faster than the solution exchange.

		Corrected pulses					Rectangular pulses			
τ_s(ms)		k_1	k_{-1}	k_2	k_{-2}		k_1	k_{-1}	k_2	k_{-2}
**1.1**	k_1	1.000	-0.208	-0.373	-0.367		1.000	0.438	0.438	-0.064
	k_{-1}	-0.208	1.000	0.945	0.010		0.438	1.000	1.000	0.461
	k_2	-0.373	0.945	1.000	0.321		0.438	1.000	1.000	0.462
	k_{-2}	-0.367	0.010	0.321	1.000		-0.064	0.461	0.462	1.000
**18.7**	k_1	1.000	0.420	-0.626	-0.749		1.000	0.637	0.637	0.259
	k_{-1}	0.420	1.000	0.092	-0.496		0.637	1.000	1.000	0.517
	k_2	-0.626	0.092	1.000	0.816		0.637	1.000	1.000	0.518
	k_{-2}	-0.749	-0.496	0.816	1.000		0.259	0.517	0.518	1.000

Supplementary Table 3 | Four correlation matrices. Currents of patches with CNGA2 channels were recorded from patch 1 (top) and patch 2 (bottom) following the complex concentration pulses shown in Fig. 5 using either rectangular or corrected concentration time courses as indicated. The currents were fitted with Model 4s4p. In the fits with rectangular pulses, k_{-1} and k_2 are strictly correlated as indicated by the correlation coefficients of 1.

	3s2p		4s2p		5s2p		6s2p	
	**value**	**SD**	**value**	**SD**	**value**	**SD**	**value**	**SD**
**k_1 ($M^{-1}s^{-1}$)**	1.62E+07	1.27E+05	3.90E+07	1.74E+05	4.85E+07	2.12E+05	5.34E+07	2.36E+05
**k_{-1} (s^{-1})**	1.03E+03	7.18E+00	3.36E+02	1.21E+00	1.92E+02	6.60E-01	1.34E+02	4.60E-01

Supplementary Table 4 | Rate constants determined for kinetic models with independent binding. The fits to complex CNGA2 current patterns are shown in Fig. 5b and the structure of the models is shown in Supplementary Table 2. The errors are given as SD.

	3s2p		4s4p		5s6p		6s8p	
	**value**	**SD**	**value**	**SD**	**value**	**SD**	**value**	**SD**
**k_1 ($M^{-1}s^{-1}$)**	1.62E+07	1.27E+05	2.13E+06	6.16E+03	8.36E+07	8.24E+07	1.08E+09	1.09E+10
**k_{-1} (s^{-1})**	1.03E+03	7.18E+00	6.51E+01	1.53E+00	5.60E+04	1.75E+04	3.75E+06	2.97E+07
**k_2 ($M^{-1}s^{-1}$)**			2.76E+08	6.29E+06	1.20E+08	1.32E+08	1.91E+08	5.60E+08
**k_{-2} (s^{-1})**			8.03E+02	4.37E+00	8.72E+00	9.14E-02	1.02E+00	2.49E-02
**k_3 ($M^{-1}s^{-1}$)**					1.54E+08	2.41E+06	6.16E+06	1.22E+05
**k_{-3} (s^{-1})**					6.53E+02	4.54E+00	2.34E+01	4.54E-01
**k_4 ($M^{-1}s^{-1}$)**							1.53E+08	3.29E+06
**k_{-4} (s^{-1})**							3.27E+02	1.68E+00

Supplementary Table 5 | Rate constants determined for kinetic models with cooperative binding allowed. The fits to complex CNGA2 current patterns are shown in Fig. 5b and the structure of the models is shown in Supplementary Table 2. The errors are given as SD.

	Patch 1 (τ_s=1.1 ms)		Patch 2 (τ_s=18.7 ms)	
	value	SD	value	SD
k_1 ($M^{-1}s^{-1}$)	1.82E+06	3.30E+03	4.41E+05	5.22E+03
k_{-1} (s^{-1})	7.67E+03	2.32E+04	1.07E+04	2.83E+05
k_2 ($M^{-1}s^{-1}$)	2.46E+10	7.44E+10	1.17E+10	3.08E+11
k_{-2} (s^{-1})	6.17E+02	1.93E+00	7.43E+01	5.50E-01

Supplementary Table 6 | Rate constants determined for the fits with rectangular pulses. The rate constants correspond to the fits shown in Supplementary Fig. 6. Notably, in both fits k_{-1} and k_2 are strictly correlated (see Supplementary Table 3) and therefore only their ratios are determined. The errors are given as SD.

Supplementary Notes

Supplementary Note 1: Quantification of ligand diffusion between pipette tip and patch
If a patch is positioned not directly at the tip of a patch pipette but in some distance within its lumen, a change of a concentration in the bath leads to a change of the concentration at the patch only after a delay because the molecules have first to diffuse from the tip to the patch. This process was quantified in the following. Both the tip opening and the patch were assumed to be equally sized circle areas forming the base and top area of a cylinder of length L. At $t < 0$, the concentration c_1 of a substance both at the pipette tip and within the cylinder are assumed to be equal and constant. At $t = 0$, the concentration of the molecule at the pipette tip, i.e. at the respective circle area, is changed in a step-like fashion, leading for an ideal step to the new constant concentration c_2 for $t > 0$. The resulting diffusion process along L is assumed to run in one dimension from $x = 0$ at the pipette tip to $x = L$ at the patch membrane.

Because the membrane patch is a barrier for the diffusing molecules, the non-binding molecules are reflected there back to the cylinder. The mathematical description of such a barrier function can be obtained by a simple trick (Supplementary Fig. 3a): The space between the pipette opening and the membrane is mirrored, leading to an additional virtual pipette opening at the same distance L to the patch membrane, but the membrane itself is taken away. The molecules diffusing from the virtual pipette opening to the membrane position and passing it then represent those molecules that are reflected by the membrane into the direction of the pipette opening.

The concentration profile $c(x,t)$ can be described by the diffusion equation

$$\frac{\partial c}{\partial t} = D\frac{\partial^2 c}{\partial x^2} \tag{S1}$$

where D is the diffusion constant. Solutions of this partial differential equation can be obtained by a separation strategy

$$c(x,t) = V(x) + T(t)\cdot X(x) \tag{S2}$$

in which V(x) and X(x) are separated location-dependent functions and T(t) is a time-dependent factor. It will become clear later from equation (S6) that $T(t)$ approximates asymptotically to zero. This leads to $V(x) = c_2 = \text{const.}$, the final concentration c_2. Inserting this in equation (S1) yields

$$\frac{\partial T(t)}{\partial t}\cdot X(x) = D\cdot T(t)\cdot\frac{\partial^2 X(x)}{\partial x^2} \tag{S3}$$

Separating time-dependent from location-dependent variables leads to

$$\frac{1}{T}\frac{\partial T}{\partial t}=\frac{D}{X}\cdot\frac{\partial^2 X}{\partial x^2}=-R \tag{S4}$$

The equality of the time- and location-dependent term for any t and x in equation (S4) can only be reached if both adopt a common constant –R.
The differential equation for the location-dependent part and its solution X(x) are now

$$D\frac{\partial^2 X}{\partial x^2}+X\cdot R=0 \qquad \Rightarrow \qquad X(x)=A\cos(kx)+B\sin(kx) \tag{S5}$$

with $k=\sqrt{\frac{R}{D}}$. Accordingly, for the time-dependent part the solution T(t) results in

$$\frac{\partial T}{\partial t}+RT=0 \qquad \Rightarrow \qquad T(t)=\exp(-Rt) \tag{S6}$$

In case of the existence of multiple pairs of solution $T_m(t)\cdot X_m(x)$ with different constants R_m (m=1, 2, …), the general solution is the linear combination of them according to

$$c(x,t)=c_2+\sum_{m=1}^{\infty}T_m(t)\cdot X_m(x)=c_2+\sum_{m=1}^{\infty}\exp(-R_m t)\cdot\left[A_m\cdot\cos(k_m x)+B_m\cdot\sin(k_m x)\right] \tag{S7}$$

with $k_m=\sqrt{\frac{R_m}{D}}$. The constants A_m, B_m can be determined by the boundary conditions at $t=0$. At this starting point, with all $T_m(0)=1$, follows

$$c(x,0)=c_2+\sum_{m=1}^{\infty}X_m(x)=c_2+\sum_{m=1}^{\infty}\left[A_m\cdot\cos(k_m x)+B_m\cdot\sin(k_m x)\right]=c_2+s(x) \tag{S8}$$

The resulting sum $s(x)$ must deliver $s(x)=c_1-c_2$ for $0<x<2L$, but at the boundaries follows $s(0)=s(2L)=0$. This leads to

$$s(x)=\sum_{m=1}^{\infty}\left[A_m\cdot\cos(k_m x)+B_m\cdot\sin(k_m x)\right]=\begin{cases}0 & \text{for} \quad x=0\\ c_1-c_2 & \text{for} \quad 0<x<2L\\ 0 & \text{for} \quad x=2L\end{cases} \tag{S9}$$

This desired process can be calculated by extending the curve to the interval $-2L \le x \le 2L$ (Supplementary Fig. 7), resulting in a rectangular shape. And the function values at the jump points, the boundaries, are zero at half the jump height. Using this convention, s(x) can be viewed as a periodic function with period 4L, which can be developed into a Fourier series with coefficients A_m, B_m.

Hence s(x) is an odd function of x, resulting in $A_m = 0 \;\; \forall m$.

In the interval of the length 4L, an integer multiple of a sinus function must fit, causing that with the possible numbers k_m for the argument of the sinus function $k_m = m\frac{\pi}{2L}$ (m = 1,2,…) and $R_m = D \cdot k_m^2 = D\frac{\pi^2 \cdot m^2}{4L^2}$ are valid.

The Fourier coefficients B_m can then be calculated according to

$$B_m = \frac{1}{2L}\int_{-2L}^{2L} s(x)\cdot \sin\left(m\frac{\pi}{2L}x\right)dx = \frac{c_1 - c_2}{L}\int_{0+0}^{2L-0} \sin\left(m\frac{\pi}{2L}x\right)dx \qquad \text{(S10)}$$

$$B_m = \begin{cases} \dfrac{4(c_1 - c_2)}{\pi m} & \text{for} \quad m = 1,3,5,7,.... \\ 0 & \text{for} \quad m = 2,4,6,8,... \end{cases} \qquad \text{(S11)}$$

When replacing m by (2n-1), the odd values of m can be expressed by the natural numbers (n = 1, 2, ….) and from equation (S7) follows

$$c(x,t) = c_2 + (c_1 - c_2)\frac{4}{\pi}\sum_{n=1}^{\infty}\frac{1}{(2n-1)}\exp\left(-D(2n-1)^2\frac{\pi^2}{4L^2}t\right)\sin\left((2n-1)\frac{\pi}{2L}x\right) \qquad \text{(S12)}$$

At the special position of the membrane (x = L) the sinus returns alternately ± 1 and we get

$$c(L,t) = c_2 + (c_1 - c_2)\frac{4}{\pi}\sum_{n=1}^{\infty}\frac{-(-1)^n}{(2n-1)}\exp\left(-D(2n-1)^2\frac{\pi^2}{4L^2}t\right) \qquad \text{(S13)} = (1)$$

For small times, many summands in equation (1) are needed. But it turned out that after about 25% of the concentration change the further time course is very close to a monoexponential time course, that can be described by the first summand only (n=1)

$$c(L,t) \approx c_2 + (c_1 - c_2)\frac{4}{\pi}\exp\left(-\frac{D\pi^2}{4L^2}\cdot t\right) = c_2 + (c_1 - c_2)\frac{4}{\pi}\exp\left(-\frac{t}{\tau}\right) \qquad \text{(S14)}$$

with the time constant $\tau = \frac{4L^2}{D\pi^2}$. (S15)

Rearranging of equations (S14) and (S15) yields

$$c(L,t) \approx c_2 + (c_1 - c_2)\exp\left(-\frac{1}{\tau}\cdot(t-t_0)\right) \qquad \text{with } t_0 = \tau\ln\left(\frac{4}{\pi}\right) \approx 0.24\tau \qquad \text{(S16) = (2)}$$

where t_0 is the starting point of the monoexponential time course with respect to the actual concentration jump (Supplementary Fig. 3b). Notably, the diffusion constant D and the distance L exclusively define the time constant τ and also the starting point t_0 of the monoexponential time course with respect to the actual time point of the concentration jump. Its value is $t_0 = 0.24\ \tau$.

A slightly adapted version of equation (2):

$$c(L,t) = \begin{cases} 0 & \text{for } t < t_o \\ c_2 + (c_1 - c_2)\exp\left(-\frac{1}{\tau}(t - t_o)\right) & \text{for } t \geq t_o \end{cases} \qquad \text{(S17) = (3)}$$

describes the kinetics sufficiently well to fit experimental time courses with typical experimental errors (inserts in Fig. 5b). On simulated data a small systematic error (<2%) was present that did not essentially affect the fit quality (Supplementary Fig. 3b).

Practically, we determined the time course of maximally open CNGA2 channels (100 µM cGMP) by changing the concentration of permeating K^+ ions from 120 mM to 150 mM. (insets in Fig. 5b). From the fitted time constant and with the diffusion constant for K^+, D_{K+} = 1.76×10^{-5} cm^2s^{-1}, we estimated the effective distance L for this patch, resulting from equation (S15). This allowed us to construct the concentration profile at the membrane for cGMP-jumps using as diffusion constant D_{cGMP}=4.4×10^{-6} cm^2s^{-1}, the value determined for the structurally closely similar cAMP [1]. Alternatively τ can be directly rescaled with the ratio of the diffusion constants, as L is assumed constant throughout a measurement.

Supplementary Note 2: Characterization of the fits including the real time courses of solution exchange

The question to be addressed is how accurate model parameters can be determined by a fit analysis of current time courses that are evoked by concentration steps of limited but known speed and profile and when these time courses are included in the fit analysis.

The following considerations were performed with the most simple gating model for a channel containing one closed state C and one open state O only,

$$\mathrm{C} \underset{k_2}{\overset{L \times k_1}{\rightleftharpoons}} \mathrm{O} \qquad \text{Supplementary Scheme 1}$$

L is the ligand concentration, k_1 the opening rate constant in $M^{-1}s^{-1}$, and k_2 the closing rate constant in s^{-1}. The rate constants are parameters in the fit. The activation time constant τ_a for this model is given by

$$\tau_a = 1 / (L \times k_1 + k_2) \qquad \text{(S19)}$$

For the solution exchange we assumed an exponential time course with the time constant τ_s. Results are shown in Figures S4 and S5.

Supplementary Note 3: Speed of the ligand step

Concerning the speed of the solution exchange determined herein, it should be noted that the total time course is composed by convection and diffusion. Convection is assumed to dominate if the membrane is outside the pipette. The contribution of diffusion is only poorly defined. One component, diffusion through an unstirred layer facing the membrane, has been suggested to last 20 μs [2], which can be ignored herein though some longer times are possible due to membrane invaginations and close packings, in particular in inside-out patches. In inside-out and outside-out patches another component of diffusion is associated with the distance to be passed between pipette opening and the patch position if they are located in the pipette lumen.

Assuming a one-dimensional diffusion situation, the mean diffusion time t_D can be estimated by

$$t_D = \lambda^2/2D \qquad \text{(S18)}$$

where D is the diffusion constant of the molecule. Along a typical distance λ=1 μm, a K^+ ion (D_K =1.96×10^{-5} cm^2s^{-1} [3] (p.268)) and a ligand of the size of ATP (D_{cAMP} = 4.4×10^{-6} cm^2s^{-1} [1]) t_D calculates to 255 μs and 1.14 ms, respectively. If λ=3 μm, the respective values for t_D are already 2.3 and 10.2 ms. Values in this range of several milliseconds were described previously to appear in outside-out patches [4].

Supplementary References

1. Dworkin, M. & Keller, K.H. Solubility and diffusion coefficient of adenosine 3':5'-monophosphate. *J Biol Chem* **252**, 864-865 (1977).
2. Sachs, F. Practical limits on the maximal speed of solution exchange for patch clamp experiments. *Biophys J* **77**, 682-690 (1999).
3. Hille, B. Ionic Channels of Excitable Membranes, 2nd ed. *Sinauer Associates Inc., Sunderland, Massachusetts*, p. 272 (1992).
4. Moffatt, L. & Hume, R.I. Responses of rat P2X2 receptors to ultrashort pulses of ATP provide insights into ATP binding and channel gating. *J Gen Physiol* **130**, 183-201 (2007).
