# Supplementary material for: Fast functional mapping of ligand-gated ion channels
Source: Commun Biol. 2023 Oct 2;6:1003. doi: 10.1038/s42003-023-05340-w (PMC10545696; doi:10.1038/s42003-023-05340-w)
Supplement: Supplementary file 4 — Reporting Summary [file 42003_2023_5340_MOESM4_ESM.pdf]

nature portfolio

Corresponding author(s): Klaus Benndorf

Last updated by author(s): Sep 4, 2023

Reporting Summary

Nature Portfolio wishes to improve the reproducibility of the work that we publish. This form provides structure for consistency and transparency in reporting. For further information on Nature Portfolio policies, see our Editorial Policies and the Editorial Policy Checklist.

Statistics

For all statistical analyses, confirm that the following items are present in the figure legend, table legend, main text, or Methods section.

n/a	Confirmed	
☐	☒	The exact sample size (*n*) for each experimental group/condition, given as a discrete number and unit of measurement
☐	☒	A statement on whether measurements were taken from distinct samples or whether the same sample was measured repeatedly
☒	☐	The statistical test(s) used AND whether they are one- or two-sided<br>*Only common tests should be described solely by name; describe more complex techniques in the Methods section.*
☒	☐	A description of all covariates tested
☐	☒	A description of any assumptions or corrections, such as tests of normality and adjustment for multiple comparisons
☐	☒	A full description of the statistical parameters including central tendency (e.g. means) or other basic estimates (e.g. regression coefficient) AND variation (e.g. standard deviation) or associated estimates of uncertainty (e.g. confidence intervals)
☒	☐	For null hypothesis testing, the test statistic (e.g. *F*, *t*, *r*) with confidence intervals, effect sizes, degrees of freedom and *P* value noted<br>*Give P values as exact values whenever suitable.*
☒	☐	For Bayesian analysis, information on the choice of priors and Markov chain Monte Carlo settings
☒	☐	For hierarchical and complex designs, identification of the appropriate level for tests and full reporting of outcomes
☒	☐	Estimates of effect sizes (e.g. Cohen's *d*, Pearson's *r*), indicating how they were calculated

Our web collection on statistics for biologists contains articles on many of the points above.

Software and code

Policy information about availability of computer code

Data collection	Data collection was done in commercial Patch-clamp software (ISO 3.0 by MFK and Patchmaster 2x90.5 bei HEKA) and in commercial microscopy software (Zen black 2010B) as supplied by the system Vendor. Control software for inhouse built Hardware is shared upon request.
Data analysis	Basic analysis and Data processing was performed in MS Excel and Wavemetrics Igor Pro (7.0.8.1). Modell fitting was performed in custom Matlab scripts, the mathematical algorithm of the scripts is provided in Supplementary Information and previous work. We are happy to share the actual implementation if reviewer or editor deem it useful.

For manuscripts utilizing custom algorithms or software that are central to the research but not yet described in published literature, software must be made available to editors and reviewers. We strongly encourage code deposition in a community repository (e.g. GitHub). See the Nature Portfolio guidelines for submitting code & software for further information.

Data

Policy information about availability of data

All manuscripts must include a data availability statement. This statement should provide the following information, where applicable:

- Accession codes, unique identifiers, or web links for publicly available datasets
- A description of any restrictions on data availability
- For clinical datasets or third party data, please ensure that the statement adheres to our policy

Raw and processed data is shared on request. As the focus is on the technique, we assume the measurement concept is of more interest than the actual data.

Human research participants

Policy information about studies involving human research participants and Sex and Gender in Research.

Reporting on sex and gender	Does not apply.
Population characteristics	Does not apply.
Recruitment	Does not apply.
Ethics oversight	Does not apply.

Note that full information on the approval of the study protocol must also be provided in the manuscript.

Field-specific reporting

Please select the one below that is the best fit for your research. If you are not sure, read the appropriate sections before making your selection.

☒ Life sciences ☐ Behavioural & social sciences ☐ Ecological, evolutionary & environmental sciences

For a reference copy of the document with all sections, see nature.com/documents/nr-reporting-summary-flat.pdf

Life sciences study design

All studies must disclose on these points even when the disclosure is negative.

Sample size	Focus is on maximizing information obtained from individual meassurement.
Data exclusions	Data with incomplete meassurments due to raptured patches and data witho bviouse technical artefacts (i.e. strong baseline drift) were excluded
Replication	Focus is on maximizing information obtained from individual measurement - threfore repetitions were minimal. Replication on the identical samples were used to show stability of the method –even under deteriorating conditions (e.g. Fig. 2). Samples with diverging physical patchposition /solution exchange were selected to exemplify the power of our diffusion correction (Fig. 5).
Randomization	Does not apply.
Blinding	Does not apply.

Reporting for specific materials, systems and methods

We require information from authors about some types of materials, experimental systems and methods used in many studies. Here, indicate whether each material, system or method listed is relevant to your study. If you are not sure if a list item applies to your research, read the appropriate section before selecting a response.

Materials & experimental systems

n/a	Involved in the study
☒	☐ Antibodies
☐	☒ Eukaryotic cell lines
☒	☐ Palaeontology and archaeology
☒	☐ Animals and other organisms
☒	☐ Clinical data
☒	☐ Dual use research of concern

Methods

n/a	Involved in the study
☒	☐ ChIP-seq
☒	☐ Flow cytometry
☒	☐ MRI-based neuroimaging

Eukaryotic cell lines

Policy information about cell lines and Sex and Gender in Research

Cell line source(s)	A HEK293 cell-lines expressing P2X2 based on the commercial T-REX System (Invitrogen) was used.
Authentication	did match functional description (antibiotica effects and protein expressed)
Mycoplasma contamination	regular testing is performed
Commonly misidentified lines (See ICLAC register)	-
